# The Largest Response Component in the Motor Cortex Reflects Movement Timing but Not Movement Type

**DOI:** 10.1523/ENEURO.0085-16.2016

**Published:** 2016-08-30

**Authors:** Matthew T. Kaufman, Jeffrey S. Seely, David Sussillo, Stephen I. Ryu, Krishna V. Shenoy, Mark M. Churchland

**Affiliations:** 1 Neurosciences Program, Stanford University, Stanford, California 94305; 2 Department of Electrical Engineering, Stanford University, Stanford, California 94305; 3 Department of Bioengineering, Stanford University, Stanford, California 94305; 4 Department of Neurobiology, Stanford University, Stanford, California 94305; 5 Cold Spring Harbor Laboratory, Cold Spring Harbor, New York 11724; 6 Department of Neuroscience; 7 Grossman Center for the Statistics of Mind, David Mahoney Center for Brain and Behavior Research, Kavli Institute for Brain Science, Columbia University Medical Center, New York, NY 10032; 8 Department of Neurosurgery, Palo Alto Medical Foundation, Palo Alto, California 94301; 9 Howard Hughes Medical Institute, Stanford University, Stanford, California 94305

**Keywords:** condition-invariant signal, dPCA, movement initiation, movement triggering, reaction time, state space

## Abstract

Neural activity in monkey motor cortex (M1) and dorsal premotor cortex (PMd) can reflect a chosen movement well before that movement begins. The pattern of neural activity then changes profoundly just before movement onset. We considered the prediction, derived from formal considerations, that the transition from preparation to movement might be accompanied by a large overall change in the neural state that reflects when movement is made rather than which movement is made. Specifically, we examined “components” of the population response: time-varying patterns of activity from which each neuron’s response is approximately composed. Amid the response complexity of individual M1 and PMd neurons, we identified robust response components that were “condition-invariant”: their magnitude and time course were nearly identical regardless of reach direction or path. These condition-invariant response components occupied dimensions orthogonal to those occupied by the “tuned” response components. The largest condition-invariant component was much larger than any of the tuned components; i.e., it explained more of the structure in individual-neuron responses. This condition-invariant response component underwent a rapid change before movement onset. The timing of that change predicted most of the trial-by-trial variance in reaction time. Thus, although individual M1 and PMd neurons essentially always reflected which movement was made, the largest component of the population response reflected movement timing rather than movement type.

## Significance Statement

The activity of neurons often conveys information about externally observable variables, such as the location of a nearby object or the direction of a reach made to that object. Yet neural signals can also relate to “internal” factors: the thoughts and computations that link perception to action. We characterized a neural signal that occurs during the transition from preparing a reaching movement to actually reaching. This neural signal conveys remarkably accurate information about when the reach will occur, but carries essentially no information about what that reach will be. The identity of the reach itself is carried by other signals. Thus, the brain appears to employ distinct signals to convey what should be done and when it should be done.

## Introduction

The responses of individual neurons are often characterized in terms of tuning: how the firing rate varies across different stimuli or behaviors (“conditions”). Additionally, neural responses may contain untuned features that are shared across many conditions, such as an abrupt rise in firing rate after the onset of any stimulus. These untuned response features may appear nonspecific, and thus of secondary interest. However, there is evidence that response features can be correlated across conditions, yet still carry computationally relevant information. Neural activity in the prefrontal cortex contains a large response component reflecting the passage of time (Machens et al., 2010), and time-varying signals have also been observed in the premotor cortex during anticipation of an informative cue (Confais et al., 2012). A related example is the time-encoding urgency signal observed during decision making, which is shared across neurons that encode different choices in both the oculomotor system (Churchland et al., 2008; Hanks et al., 2011) and the premotor cortex (Thura et al., 2012). Here we investigate another possible “untuned” signal in the motor/premotor cortex: one that arises after the desired target is known, at the time of the sudden transition from preparation to movement.

We were motivated by the observation that motor cortex neurons sometimes display broadly tuned movement-period responses (Fortier et al., 1993; Crammond and Kalaska, 2000)*—*e.g., a rise in rate for all directions*—*such that tuning models benefit from an omnidirectional term (Georgopoulos et al., 1986; Moran and Schwartz, 1999). More generally, many studies identify significant proportions of neurons with responses that are task-modulated yet not strongly selective for the parameter being examined (Evarts, 1968; Weinrich et al., 1984; Hocherman and Wise, 1991; Riehle et al., 1994; Messier and Kalaska, 2000). These findings argue that there must be some aspect of neural responses*—*i.e., some response “component”*—*that is at least moderately correlated across conditions. What are the temporal properties of such a signal and is its timing predictive of behavior? Does the signal make a small or large contribution to the overall population response? Is the signal merely correlated across conditions (“condition-correlated”)? Or might it be nearly identical across conditions (“condition-invariant”) and thus untuned in the traditional sense?

These questions derive both from a general desire to fully characterize the response during movement and from specific theoretical considerations. A condition-invariant signal (CIS) could, despite its seeming lack of specificity, be important to the overall computation performed by the population. Presumably there is a large change in computation just before movement onset, at the moment when the motor system transitions from preparing to move while holding a posture (Kurtzer et al., 2005) to generating the muscle activity that will drive the desired movement. Consistent with the idea of a change in computation, neural tuning changes suddenly and dramatically at a point ∼150 ms before movement (Churchland et al., 2010) so that a neuron’s “preference” during movement can be quite unrelated to its preference during preparation (Wise et al., 1986; Crammond and Kalaska, 2000; Kaufman et al., 2010). A similar transition is observed at the population level: population dynamics are relatively stable and attractor-like during preparation but become strongly rotational just before movement onset (Churchland et al., 2012). This sudden change in network properties is presumably driven by an appropriately timed input (which could itself be the output of a computation that decides when to move; Romo and Schultz, 1987; Thaler et al., 1988; Schurger et al., 2012; Murakami and Mainen, 2015). One might initially expect a “triggering” input to be tuned (Johnson et al., 1999; Erlhagen and Schöner, 2002). Yet theoretical considerations suggest that a simple, condition-invariant change in input is sufficient to trigger large changes in network dynamics and tuning (Hennequin et al., 2014). In particular, a recent neural network model of motor cortex (Sussillo et al., 2015) uses a condition-invariant input to trigger a change in dynamics that initiates movement. The model’s population-level responses resemble the empirical neural responses, and from inspection both clearly show at least some features that are invariant across conditions.

Critically, there are many ways in which activity patterns can be correlated across conditions. Only a minority of such possibilities involve a true CIS at the population level: that is, a signal that is nearly identical across conditions. Is a CIS present in motor cortex? On a trial-by-trial basis, does it exhibit timing locked to target onset, the go cue, or movement onset? Only the latter would be consistent with the role in movement triggering suggested by the model of Sussillo et al. (2015).

We found that a CIS was not only present but was the largest aspect of the motor cortex response*—*considerably larger than any of the condition-specific (tuned) response components. The CIS resembled the previously reported omnidirectional or “speed-tuned” response component (Georgopoulos et al., 1986; Moran and Schwartz, 1999), but was essentially invariant with reach speed, distance, and curvature. In addition, the CIS underwent a large and sudden change ∼150 ms before movement onset. The timing of this change was an excellent predictor of reaction time (RT) on a trial-by-trial basis. Finally, the dimensions in neural state space that were occupied by the CIS were almost perfectly orthogonal to the dimensions occupied by the condition-specific components. Overall, the profile, timing, and population-level manifestation of the CIS were remarkably similar to the structure naturally produced by the model of Sussillo et al. (2015). Our findings thus suggest a potential role for a large response component that initially appears nonspecific yet reflects movement timing very precisely.

## Materials and Methods

The key features of the task and analyses are described in the Results. Below we detail all aspects of the apparatus, task, neural recordings, muscle recordings, data preprocessing, analyses, and controls.

### Subjects and task

Animal protocols were approved by the Stanford University Institutional Animal Care and Use Committee. Experiments employed two adult male rhesus monkeys (*Macaca mulatta*), J and N, performing a delayed-reach task on a frontoparallel screen (Churchland et al., 2010, 2012; Kaufman et al., 2013). The monkey initially fixated a central spot with his eyes and touched it with a cursor. The cursor was projected slightly above the right fingertip, which was tracked optically. The task involved a large number of conditions*—*i.e., different target locations and reach paths*—*which was useful when attempting to identify response components that are invariant across conditions. On one-third of trials (“no-barrier” conditions) a lone target appeared within a frame around the workspace. On another one-third of trials (“maze” conditions) a target and ≤9 virtual barriers appeared. The remaining one-third of trials (“maze-with-distractor” conditions) were identical to the maze trials but included two distractor “targets” that were unreachable due to the barrier locations. The same set of target positions was used for the no-barrier, maze, and maze-with-distractor conditions. When barriers were present, the monkey had to perform a curved reach or the cursor would collide with and “stick” to the barrier. This paradigm evoked both straight and curved reaches in different directions and of varying speed and distance. Most datasets employed 27 conditions (nine of each type) while one (NAC) employed 108. No attempt was made to produce a uniform arrangement of target locations or initial reach directions, but we note that all datasets involved reaches that spanned the space of directions in two-dimensional space, and that results were consistent across the different datasets, which typically employed different arrangements of targets and barriers. More broadly, the large variety of conditions we employed provides a stringent test regarding whether a signal is truly condition-invariant.

A randomized delay period separated target onset from a go cue. During the delay, targets jittered slightly (2–3 mm), indicating to the monkey that he could not yet reach or saccade. The go cue consisted of three simultaneous and salient cues: jitter ceasing, the targets changing from open to filled, and the central spot disappearing. Juice reward was delivered if the monkey swiftly reached to the target then held it for 450 ms (monkey J) or 700 ms (monkey N).

### Delay-period statistics

The delay period lasted 0-1000 ms. Different datasets employed different delay-period statistics, depending on the analyses we wished to apply. Three datasets (JC, NAC, and NS) were collected with the primary goal of analyzing trials with longer delays. Longer delays enabled examination of the transition between a relatively stable plateau of preparatory activity and subsequent movement-related activity. To this end, delays of 450-1000 ms were approximately twice as probable as delays of 0-450 ms. Three further datasets (JAD1, JAD2, NAD) were recorded with the goal of characterizing the single-trial relationship between neural activity and response time (RT). For these datasets, delay durations of 0, 100, 200, and 500 ms were intentionally over-represented. These dataset names end with “D,” indicating that this set of discrete delays was over-represented. This allowed key analyses to be restricted to a set of trials with the same delay, removing the potential confound that RT can vary with delay. For these datasets most trials (78, 78, and 84% for datasets JAD1, JAD2, and NAD) used one of the discrete delays, with roughly equal probability. The remaining trials had random delays from 0 to 1000 ms as above. Because these datasets were each collected in a single day using implanted multielectrode arrays, the monkeys were not anticipating the over-represented delay durations.

Most analyses focused on the transition from movement preparation to movement and thus used only trials with >450 ms delays (datasets without discrete delays) or 500 ms delays (datasets with discrete delays). For analyses of the single-trial relationship with RT, we focused on datasets with discrete delay durations. For simplicity of presentation, for these analyses only trials with no delay (“zero delay”) or a 500 ms delay (“long delay”) are shown. All results were similar for delays of 100 or 200 ms.

### Catch trials and trial counts

Several types of unanalyzed catch trials ensured the task was performed as desired. In particular, we presented novel mazes made by randomly removing barriers from a standard maze (10–15% of all trials), or randomly placing the target and two barriers (0–10% of all trials). These trials ensured that the monkey had to solve each trial independently, as similar-looking mazes could have different solutions.

Delay periods were randomly chosen on each trial. Conditions were organized in pseudorandom blocks. The array datasets had 3352, 2340, 2622, and 3590 successful trials (datasets JAD1, JAD2, NAD, and NAC) from a single session. For the “discrete delay” datasets (JAD1, JAD2, and NAD) there were ∼250–500 usable trials for each of the four over-represented delays. Usable trials excluded catch trials, failed trials (e.g., if a barrier were struck), rare trials with an unusual velocity profile that did not allow a reliable RT measurement, and trials with a very short RT (in rare instances where the monkey “jumped the gun”) or an overly long RT (in rare instances where the monkey was presumably distracted). Datasets that included single-unit recordings (JC and NS) contained an average of 336 and 305 usable trials per unit.

### Neural and muscle recordings

For both monkeys, we first performed single-electrode recordings (datasets JC and NS) using moveable tungsten microelectrodes (Frederick Haer) and a Plexon Multichannel Acquisition Processor (Plexon). These recordings included the caudal portion of the dorsal premotor cortex (PMd) and both surface and sulcal primary motor cortex (M1). All units recorded with single electrodes were well-isolated single neurons recorded from regions where microstimulation produced movement of the arm (typically the upper arm and/or shoulder). Each monkey was then implanted with two 96-electrode silicon arrays (Blackrock Microsystems), located in M1 and caudal PMd, as estimated from anatomical landmarks and previous mapping with microstimulation. Spikes were sorted off-line using custom software (MKsort, https://github.com/ripple-neuro/mksort). For array recordings, both single units and stable multiunit isolations (typically two neurons whose spikes could not be reliably separated) were analyzed. A strong CIS (see below) was present regardless of whether a dataset involved pure single-unit isolations or a mixture of single-unit and multiunit isolations. This is unsurprising: dimensionality reduction techniques, such as demixed principal component analysis (dPCA) or PCA, typically produce nearly identical results regardless of whether isolations involve one unit or a few units. These techniques are forgiving because the components needed to compose the responses of a single neuron are the same components needed to compose the summed response of >1 neuron. All neural recordings were from the left hemisphere. Array recordings produced datasets JAD1, JAD2, NAD, and NAC, and were included in dataset JC.

We analyzed all units where the firing rate range (over conditions and times) was greater than the maximal SEM (for all conditions and times). This signal-to-noise (SNR) criterion does not insist on any particular form of response or tuning—only that there be some response. For dataset JAD1, 116 of 123 units passed the SNR criterion; for dataset JAD2, 136 of 171 units passed; for dataset JC, 186 of 278 units passed; for dataset NAD, 172 of 188 units passed; for dataset NAC, 213 of 223 units passed; for dataset NS, 118 of 118 units passed. Of these, 67, 28, 108, 62, 58, and 118 were considered single units (datasets JAD1, JAD2, JC, NAD, NAC, and NS). For all analyses, results were similar when data from PMd and M1 were analyzed separately. These recordings were therefore pooled.

Data preprocessing involved three steps. First, spike trains were smoothed with a Gaussian (28 ms SD). Second, the firing rate was averaged across trials of the same type (excepting analyses of single trials; see below). We computed two averages: one with data aligned to target onset and one with data aligned to movement onset. Third, the firing rate of each neuron was normalized to prevent analyses from being dominated by a few high-rate neurons; this is especially important (Yu et al., 2009) when performing PCA-based analyses. To normalize without overamplifying the greater noise associated with low firing rates, we “soft normalized”: for each neuron we normalized the firing rate by its range (across all times and conditions) plus a constant, chosen to be 5 spikes/s. This choice follows our previous work, and was made before performing analyses. Results were extremely similar and sometimes stronger if we used a soft-normalization constant of zero.

Electromyographic (EMG) recordings used hook-wire electrodes (44 gauge with a 27 gauge cannula; Nicolet Biomedical), inserted percutaneously into the muscles of the right arm. Electrodes were inserted with the monkey awake and calm, with one recording per session. For monkey J, recordings were made sequentially from trapezius, latissimus dorsi, pectoralis, triceps brachii, medial and lateral aspects of the biceps brachii, and anterior, medial, and posterior aspects of the deltoid. The recording from the triceps was excluded because it was not sufficiently modulated during the task. For monkey N, recordings were made from proximal, middle, and distal aspects of the trapezius, latissimus dorsi, pectoralis, triceps brachii, medial and lateral aspects of the biceps, and anterior, medial, and posterior aspects of the deltoid. Two recordings were made for each deltoid site. The recordings from the triceps and latissimus dorsi were excluded because they were not sufficiently modulated during the task. Raw EMG signals were band-pass filtered (150–500 Hz, four pole, 24 db/octave), differentiated, rectified, smoothed with a Gaussian (15 ms SD), and averaged across trials (Kaufman et al., 2013).

### Projections of neural data

We identified response components by projecting the population response onto dimensions of interest. We began with a matrix, *R*, of trial-averaged neural responses (or EMG, for one analysis). Each of *n* columns contained the normalized response of one neuron over time, with responses concatenated across conditions. To project the data onto a given dimension we computed $\vec{x}=R\vec{w}$, where $\vec{w}$ is a set of weights specifying the dimension. The projection $\vec{x}$ is therefore a weighted average of neurons’ firing rates. We refer to the projected activity pattern as a “component” of the population response, because the activity of any given neuron can be (approximately) composed of a weighted sum of multiple such components. This use of the term “component” follows the usage of Kobak et al. (2016) and others. Note that this use of “component” is not synonymous with “principal component,” which refers to a component of the neural covariance matrix and thus corresponds to a neural dimension.

Many studies have been concerned with how to best find projections given different goals and hypotheses. In this study the most important projection method uses dPCA (Machens et al., 2010; Brendel et al., 2011) to find the dimensions $\vec{w}$. This application of dPCA is detailed more thoroughly in the next section.

We also use a number of other projection methods, including standard PCA, and simply computing the mean across neurons (equivalent to setting all weights to 1/*n*). Two analyses use the jPCA method (Churchland et al., 2012), and in one case we used a classifier trained via a supervised algorithm. In every case it should be stressed that the projections shown (i.e., the response components) are simply linear weightings of the recorded neural responses. The use of multiple methods is desirable because no single method can capture all aspects of the response (e.g., the mean captures some aspects of the response and hides others).

All projection methods used here employ orthonormal dimensions. The orthogonality of these dimensions does not impose orthogonality on aspects of the neural response; it is simply a way of choosing a coordinate system. An orthonormal basis makes interpretation simpler: among other benefits, it allows each component to be independently quantified in terms of variance explained, making it harder to unintentionally interpret weak structure as meaningful. In all cases, when a percentage of variance is quoted, it is the fraction of the variance captured in the low-dimensional space (10–12 dimensions).

### Identifying the CIS via dPCA

Many of our central analyses sought to determine whether there exist neural dimensions that segregate condition-specific (“tuned”) components from condition-invariant components of the population response. By “condition-specific” we mean that different conditions (reach directions, curvatures, etc.) evoke different responses when the population response is projected onto that dimension.

By “condition-invariant” we mean that the response varies with time but is similar across conditions when projected onto that dimension. To address this question, we applied dPCA (Machens et al., 2010; Brendel et al., 2011), a variant of PCA. dPCA leverages information normally discarded by PCA: each row of the data matrix *R* is assigned labels. Here, those labels indicated the condition and time for which that set of firing rates was recorded. dPCA then finds a matrix W that produces a projection X of the data R, with X=RW. Each column of W is a dimension and each column of X is a component of the population response. Like PCA, dPCA attempts to find a projection that captures much of the variance in R, so that $R\approx XW^{T}.$ Unlike PCA, dPCA attempts to find W such that the resulting columns of X covary strongly with one label or the other. In the present case, dPCA attempts to find W such that some columns of X (some components) vary with time but not condition and other columns vary across conditions but not with time. As will be discussed below, such segregation is not necessarily possible: in general there will not exist a W with the desired properties. Indeed, in the present study, dPCA always found components that varied primarily with time (and not condition) but never found components that varied primarily with condition and not time. We therefore divided the components found by dPCA into two groups: condition-invariant (reflecting primarily time) and condition-specific (reflecting both condition and time). We refer to the group of condition-invariant components collectively as the CIS.

As a technical note, dPCA (unlike PCA) requires that the number of dimensions be specified in advance. Prior analyses indicate that 6–8 dimensions capture much of the condition-specific structure of the data (Churchland et al., 2010). We therefore wished that dPCA should capture a similar amount of condition-specific structure, in addition to any condition-invariant structure that might be present. We empirically picked the number of requested dimensions such that dPCA returned eight condition-specific dimensions (defined as containing <50% condition-invariant variance). In principle this might have necessitated requesting exactly eight dimensions (if all structure were tuned) or many more than eight (if little structure were tuned). In practice it was only necessary to request modestly more than eight total dimensions. For example, for dataset JAD1 we requested 10 total dimensions, which yielded two condition-invariant response components and eight condition-specific response components. The choice of eight condition-specific components is an arbitrary but reasonable cutoff. We always found a strong CIS regardless of the exact number of dimensions requested. dPCA identified dimensions (W) based on the population response from −200 to +400 ms relative to target onset and −300 to +600 ms relative to movement onset. The data matrix being analyzed contained trial-averaged firing rates for long-delay trials (trials with delay periods >450 ms). For subsequent analyses of trial-to-trial variability in RT, we projected data from individual trials, including zero-delay trials, onto the same dimensions. The probabilistic-model version of dPCA was used (from the Python code available online associated with Brendel et al., 2011). We measured for each response component the marginal variances (Machens et al., 2010; Brendel et al., 2011), which indicate how much of a component’s variance was condition-specific (activity varying with condition or with both time and condition) versus condition-invariant (activity varying with time alone).

Because EMG responses were lower dimensional than neural responses, for the EMG datasets dPCA was performed at an overall dimensionality that returned three condition-specific dimensions. The resulting 4–5 dimensions (monkey J, N) accounted for 95–97% of the total variance in the EMG data. This reduced number of dimensions did not produce the differences between neural and muscle data: repeating the analysis on neural data using 4–5 dimensions yielded essentially identical results to those obtained with more dimensions.

### Note regarding interpretation of the segregation produced via dPCA

Below we describe a key interpretational point regarding the dPCA method. The cost function optimized by dPCA attempts to find W such that each column of X (each response component) varies with exactly one of the provided labels (time and condition in this study) and not with the other(s). Yet as stated above, this segregation is not in general possible. In the present case, this has two implications. First, it is not guaranteed that dPCA will be able to find components that vary with condition but not with time; perhaps every component that strongly reflects condition also reflects time (this was indeed true of our data). Second, it is similarly not guaranteed that dPCA will be able to find components that vary with time but not condition; it may be that every component that strongly reflects time also reflects condition.

This last fact is worth stressing because many individual neurons exhibit what we refer to as “condition-correlated” structure: responses that are different across conditions, yet display an increase (or decrease) in firing rate that has a somewhat similar time course across conditions. Yet this structure at the single-neuron level is not sufficient, in and of itself, to indicate condition-invariant structure at the population level. Would dPCA, when applied to a population of such neurons, inevitably find condition-invariant components? In short, it would not. This can be demonstrated empirically (see Results) or formally via construction, as follows. Consider a simple case in which each neuron’s response *r_n_* is a linear combination of two independent components $x_{i}$ (which will also be functions of condition *c* and time *t*): $r_{n,c,t}=\underset{i=1:2}{\sum }w_{n,i}\,x_{i,c,t}$. Let both $x_{1,c,t}$ and $x_{2,c,t}$ be condition-specific, but suppose $x_{1,c,t}$ contains an overall correlation between conditions. Due to the correlation of $x_{1,c,t}$ across conditions, the responses *r* will also have shared response features across conditions. Nevertheless, it is not in general possible to find a linear combination of the $r_{n,c,t}$’s that is condition-invariant. A linear combination of the $r_{n,c,t}$’s is equivalent to a linear combination of $x_{1,c,t}$ and $x_{2,c,t}$. Since these components are independent, finding a condition-invariant linear combination is equivalent to solving the following system of $\left(C-1\right)T$ equations, where *C* is the number of conditions (here, 2), and *T* is the number of time points:

$$\underset{i=1:2}{\sum }p_{i}x_{i,c,t}=\underset{i=1:2}{\sum }p_{i}x_{i,c+1,t}$$

for all times *t* and all pairs of conditions *c* and *c* + 1 (this is a sufficient constraint to ensure that all pairs of conditions are equal, since equality is transitive).

The number of free variables $p_{i}$ is equal to the number of components *D*, which in this example is 2. In general, then, this system is not solvable if $\left(C-1\right)T>D$, which will be true for even modest numbers of times and conditions. The presence of correlated structure within $x_{1,c,t}$ (and/or $x_{2,c,t}$) would not in general change this fact. In practice, then, it would be rare for condition-correlated responses to coincidentally produce a fully condition-invariant component. As one example, choose $x_{1,c,t}{=g}_{c}sin(t)$ and $x_{2,c,t}\;{=h}_{c}sin(3t)$, with $g_{c}$ and $h_{c}$ being positive scalars that vary with condition. Both$\;x_{1,c,t}$ and $x_{2,c,t}$ would be perfectly condition-correlated, yet no linear combination of $x_{1,c,t}$ and $x_{2,c,t}$ would be condition-invariant.

### Control: producing synthetic peristimulus time histograms with matched spectral content

To illustrate empirically that condition-invariant components are not found in “generic” data, we generated synthetic peristimulus time histograms (PSTHs) with the same frequency content as the original neurons. Each unit was matched with a corresponding synthetic PSTH. The steps below were performed on the vector containing the trial-averaged firing rate over time for one condition. We first preprocessed each vector by smoothing lightly (10 ms SD Gaussian) to reduce the small discontinuity between target-aligned and movement-aligned data, then multiplying by a Hann window. The Fourier transform was performed, and the magnitude of the result was computed at each frequency (i.e., the square root of power spectral density). These curves were averaged over conditions to give the overall power-by-frequency curve for that unit. To construct a synthetic PSTH for each condition, we chose a random phase for each frequency component, then took the inverse Fourier transform.

### Control: removing the CIS from the neural responses

To ask whether condition-invariant components (collectively the CIS) might result from the rectification of firing rates at zero, we removed the true condition-invariant components, rerectified firing rates, then applied dPCA. Specifically, we projected the population response onto the eight condition-specific dimensions identified by dPCA, then transformed the data back to the original *n*-dimensional space. This produced as many PSTHs as the original neurons. We rescaled and recentered each “neuron’s” response to restore its original mean and range of firing rates. Finally, we set all negative firing rates to zero. This resulted in a population of surrogate neurons that are responsive and have positive firing rates, yet should have no “true” CIS. Thus, a strong CIS in this control population would indicate that rectified firing rates could create an artifactual CIS.

### Control: adding a condition-correlated component

We constructed additional surrogate data that resembled the empirical data but lacked condition-invariant components. For each empirical condition-invariant component, we constructed a new component with the same time course, but with varying amplitude across conditions. That is, we created components that were condition-correlated but not condition-invariant. These components were recentered to have a zero mean during the baseline period (before target/maze onset), and then were added to the response of each neuron. Specifically, to each neuron’s response *r_n_*_,_*_c_*_,_*_t_* we added *w_n_*_,_*_i_k_c_x’_i_*_,_*_t_*, where *w_n_*_,_*_i_* is the neuron’s original weight for the *i* ^th^ condition-invariant component, *x’_i_*_,_*_t_* is the time course of the *i* ^th^ new condition-correlated component, and the coefficients *k_c_* were chosen randomly from a unit-variance Gaussian distribution. We rectified the resulting firing rates (setting all negative rates to zero). These operations largely preserved the time course of each neuron’s across-condition mean (because the *k_c_*’s were zero-mean). Because the new components were condition-correlated, the responses of most neurons were strongly condition-correlated. Yet because the original condition-invariant components are now “contaminated” with condition-specific components of the same time course, the surrogate population should have no separable condition-invariant components.

### Identifying a speed-predicting dimension

To identify a speed-predicting dimension, we began with the same neural data matrix *R* used for PCA and dPCA. We then regressed the trial-averaged speed profile for each condition against *R*: $\vec{s}=R\vec{w}+b$, where $\vec{s}$ is the vector produced by taking the speed profile for each condition and concatenating the conditions, *b* is the bias (a constant offset), and $\vec{w}$ specifies a set of weights. The speed profile was advanced by 150 ms before regression to accommodate known lags.

### Trial-by-trial analysis

To assess how well projections onto different dimensions predict trial-by-trial movement onset we performed four steps: (1) we chose a potentially informative weighted sum of neurons (“dimension of interest”); (2) we binned and smoothed the spiking data on individual trials; (3) we projected the population neural response from each trial onto the dimension of interest; and (4) for each trial we found the time point at which that projection exceeded a criterion value. That time, relative to the go cue, was the predicted RT. These steps are explained in more detail below.

For the first step, we compared the performance of several different techniques for finding the dimension of interest. Three of these techniques were unsupervised: dimensions were identified based on the structure of the data without exploiting prior knowledge of the RT. These three methods—the CIS_1_ method, the PC_1_ method, and the mean-over-all-neurons method—used dPCA, PCA, and simple averaging, respectively. The CIS_1_ dimension (producing the largest condition-invariant component) and the PC_1_ dimension (the largest principal component) were found using the long-delay, trial-averaged data (as above). We also employed a linear decoder of reach speed (see above) and a supervised “classifier” method, described below.

For the second step, spikes were counted in 10 ms bins, from 60 ms before to 500 ms after the go cue. Each trial’s spike counts were convolved with a 30 ms Gaussian to produce a smooth spike rate. For the third step, we computed a weighted sum of the neurons’ spike rates. The weights depended on the dimension of interest, found during step one. We refer to the result of this third step as *z*(*t,r*), the projection of the neural data as a function of time and trial.

For the final step, we wished to determine when *z*(*t,r*) changed in advance of movement onset. To estimate that time, for each trial we asked when *z*(*t,r*) first crossed a criterion value derived from the long-delay trials. To find that criterion value, we took the median of *z*(*t,r*) across trials, producing $\overset{˜}{z}(t)$. We set the criterion value to be the midpoint of $\overset{˜}{z}\left(t\right)$: $[\max\left(\overset{˜}{z}\left(t\right)\right)+\min\left(\overset{˜}{z}\left(t\right)\right)]/2$. The midpoint is an arbitrary but reasonable choice to ensure robustness. For each trial, we found the time at which the criterion value was crossed. Trials that never exceeded the criterion value, or that exceeded it before the go cue, were discarded from the analysis. Such trials were uncommon, especially for the better prediction methods (0–9%, depending on dataset and method).

The three methods described above—the CIS_1_ method, the PC_1_ method, and the mean-over-all-neurons method—predict RT in an unsupervised manner. They were compared with a supervised method that was allowed to use knowledge of each trial’s RT. This “classifier” method was based on logistic regression. Single-trial data were first aligned to movement onset, then projected into the dPCA space (including both condition-specific and condition-invariant dimensions). Data were binned into a “premovement” time point (−360 to −150 ms relative to movement onset) and a “movement” time point (−150 to +60 ms relative to movement onset). The dividing point of 150 ms before movement onset was chosen to approximate the delay between when neural firing rates begin to change and when the hand begins to move. Logistic regression returned both a projection dimension and a criterion value that best discriminated between the premovement and movement data.

As with the other projection methods, the classifier produces a projection vector $\vec{w}$ with as many coefficients as dimensions of the data (in this case, the number of components from dPCA). To characterize the classifier, we asked how much each dPCA component contributed to this projection. Specifically, we took the quantity $\left|{\vec{w}}_{d}\right|\cdot \sqrt{var[{\left(RD\right)}_{d}]}$, where $\left|{\vec{w}}_{d}\right|$ is the absolute value of the *d* ^th^ element of $\vec{w}$, *D* is the dPCA projection matrix (called *W* in previous equations), (*RD*)*_d_* indicates the *d* ^th^ column of the matrix resulting from multiplying *RD*, and *var*[] indicates taking the variance. This tells us how strongly each of the response components (returned by dPCA) contributed to the final classification.

Finally, we used a semisupervised method where RT was predicted as the time when the decoded reach speed crossed a 50% threshold. Importantly, for all the above methods, training employed only the long-delay data. Trial-by-trial prediction of RT for zero-delay data was entirely based on generalization. Analyses were based on 385/465 trials for dataset JAD1 (long-delay/zero-delay), 249/264 trials for dataset JAD2, 260/427 trials for dataset NAD, and 2982 long-delay trials for dataset NAC.

### Finding a rotational plane

For some analyses, we wished to identify planes (two-dimensional projections of the population response) containing rotational structure. We performed dPCA and then applied jPCA (Churchland et al., 2012) to the condition-specific components, using an epoch when neural activity is changing rapidly (−200 to +150 ms relative to movement onset). As a technical detail, the PCA step and mean subtraction were disabled in the jPCA algorithm; dPCA served as a more principled way of focusing jPCA on the strongly condition-specific dimensions. Because both dPCA and jPCA produce linear projections, the final result is also a linear projection of the data.

## Results

### Behavior and neural recordings

**Figure 1.**
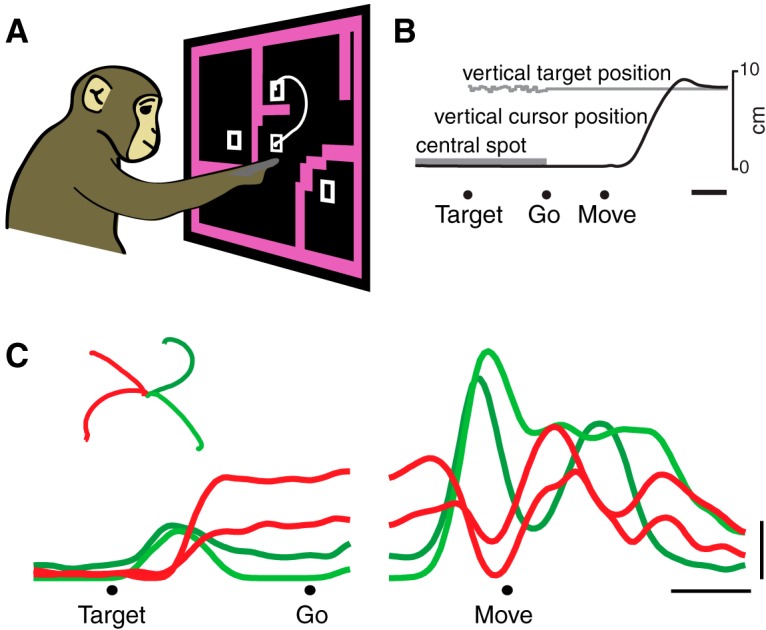
Task and basic neural responses. ***A***, ***B***, Illustration of the maze task. Monkeys executed reaches that avoided any intervening barriers. The task was performed with a cursor presented just above the monkey’s hand. White trace shows the path of the cursor on one trial. Target, Target onset; Go, go cue; Move, movement onset. ***C***, PSTH for an example neuron for four (of 27) conditions. Each trace shows the trial-averaged firing rate for one reach condition (one unique maze) over time. Averaging was performed twice: locked to target onset (left traces) and movement onset (right traces). Only trials with a 500 ms delay were included. Inset, Reach trajectories, colored the same as their corresponding neural traces. This neuron illustrates the transition between stable preparatory activity and rapidly changing movement-related activity. Scale bars: ***B***, ***C***, horizontal, 200 ms; ***C***, vertical, 10 spikes/s.

Two monkeys (J and N) performed a variant of the standard delayed-reaching task: the maze task (Fig. 1*A*,*B*; Churchland et al., 2010, 2012; Kaufman et al., 2013). The monkey touched and fixated a central spot on a screen, then was presented with a target and, on most trials, a set of virtual barriers (magenta rectangles). After a randomized delay period, a go cue was presented, and the monkey was required to reach to the target, curving around barriers if present. We refer to each target/barrier configuration as a “condition.” RTs were brisk: medians of 296 ms (monkey J) and 304 ms (monkey N).

We analyzed six datasets. Three datasets (JAD1, JAD2, and NAD) were collected specifically for this study. For these, recordings were from a single session, made via a pair of 96-electrode arrays, one in PMd and one in M1. To ensure robustness, we also reanalyzed three datasets that have been previously examined. One (NAC) was recorded using a pair of 96-electrode arrays, one (NS) was recorded over many days using single electrodes, and one (JC) combined 1 day of array recordings and many days of single-electrode recordings. These latter two datasets enabled us to analyze large populations that contained both surface PMd/M1 recordings and sulcal M1 recordings.

**Figure 2.**
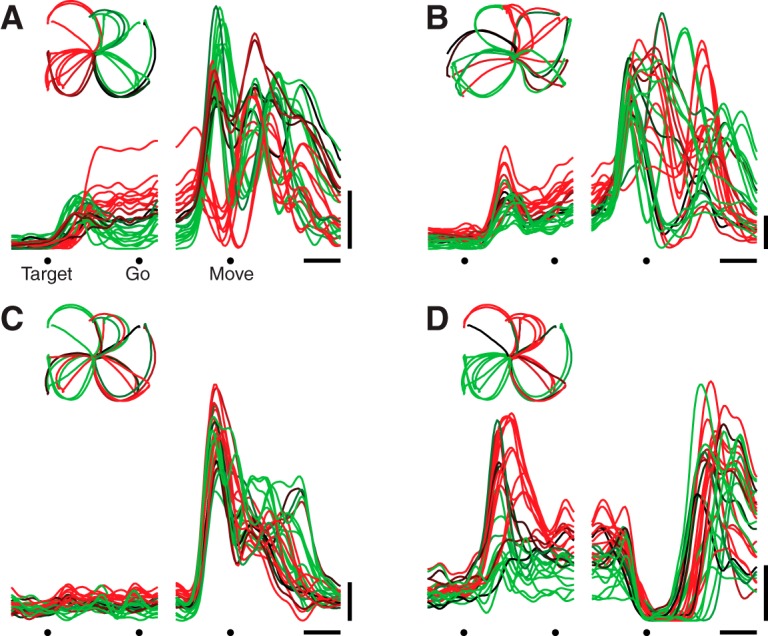
Responses of four example neurons. Format is as in Figure 1*C*, but responses are shown for all 27 conditions. ***A***, Unit with complex responses. This neuron showed both an overall increase in firing rate across conditions and a strong oscillatory component that was condition-specific (unit JAD1-98, same as in Fig. 1*C*). Scale bars same as Figure 1*C*. Inset in upper left shows reach trajectories, colored the same as their corresponding neural traces. ***B***, Another unit with complex condition-specific responses, recorded from the other monkey (unit NAD-165). ***C***, Unit with responses that were strongly condition-correlated (unit JAD1-70). ***D***, Unit where the initial response was condition-correlated: a decline across all conditions. Later activity is more condition-specific (unit JAD1-114).

The firing rate versus time of a representative neuron is illustrated in Figure 1*C* (for ease of visualization, 4 of 27 conditions are shown). The neuron began responding ∼50 ms after target onset, and achieved different firing rates, depending on which reach the monkey was preparing (Tanji and Evarts, 1976; Weinrich et al., 1984; Godschalk et al., 1985; Kurata, 1989; Riehle and Requin, 1989; Snyder et al., 1997). Firing rates plateaued during the delay period, changing little until after the go cue. Approximately 150 ms before movement onset, there was a large transition in the response pattern: activity subsequently evolved in a seemingly complex fashion, producing a series of peaks and valleys. Such features were not due to sampling error but were very reliable (SEs of the firing rate were ∼2 spikes/s, compared to the overall firing-rate range of ∼45 spikes/s). The pattern illustrated in Figure 1*C* was typical: most neurons showed a relatively stable plateau of tuned preparatory activity followed by temporally complex responses. The relevant transition occurred just before movement onset. The response of this neuron across all 27 conditions is plotted in Figure 2*A*. Figure 2*B* plots the response of another example neuron with complex multiphasic responses that varied strongly across conditions.

The complexity and heterogeneity of responses makes it difficult to ascertain whether there might exist an underlying signal shared across reaches of different types. However, we did occasionally observe neurons where, following the go cue, the response was similar across conditions: i.e., an overall increase or decrease in rate (Fig. 2*C*,*D*). This observation is consistent with the utility of including an omnidirectional component when fitting tuning curves (Georgopoulos et al., 1986; Moran and Schwartz, 1999). More generally, the presence of such neurons is consistent with many prior reports in which some reasonable percentage of neurons were modulated by the task yet not strongly selective for the parameter being tested: e.g., left versus right reaches (Weinrich et al., 1984), three curvatures (Hocherman and Wise, 1991), two or three distances (Riehle et al., 1994; Messier and Kalaska, 2000), or two loads (Evarts, 1968). The present results demonstrate that prior findings were not a trivial result of using few conditions. We employed 27 conditions (108 for dataset NAC) spanning different directions, distances, and reach curvatures, yet still found neurons whose responses were similar across all conditions. Nevertheless, we stress that while individual neurons often showed related structure across conditions—i.e., they were condition-correlated—they essentially never showed fully condition-invariant responses. For example, even the neuron in Figure 2*C*, which has unusually strong condition-correlated structure, displayed peak firing rates that differed between conditions by a factor of nearly two.

### Population-level structure

Given that single neurons can exhibit condition-correlated responses, some underlying population-level component must be correlated across conditions. To appreciate how this can happen, consider the standard model in which each neuron’s response is a weighted sum of population-level components. The response *r* of neuron *n* at time *t* for condition *c* is as follows (Eq. 1):

$$r_{n,c,t}=\underset{i}{\sum }w_{n,i}x_{i,c,t}$$

where $x_{i,c,t}$ is the *i* ^th^ response component (one element of the population state $x_{c,t}$) and $w_{n,i}$ determines the contribution of component *i* to the response of neuron *n*. A component is “condition-correlated” if $corr\left(x_{i,c_{j},:},\;{\;x}_{i,c_{k},:}\right)$ is positive when averaged across all choices of conditions $c_{j}$ and $c_{k}.$

The possible presence of a condition-correlated component has been considered in many contexts: e.g., decision variables are often modeled as reflecting evidence for a choice (which differs across conditions) plus a growing urgency to make some choice (which is shared across conditions; Churchland et al., 2008; Hanks et al., 2011; Thura et al., 2012; Thura and Cisek, 2014). In the case of reaching, many models include a nondirectional term reflecting hand speed (Georgopoulos et al., 1986; Moran and Schwartz, 1999). Since speed is always positive, and is by definition time-locked to movement onset, a component that reflects speed will be strongly condition-correlated.

In general a condition-correlated component can vary strongly across conditions; the temporal profile must be similar but the amplitude can vary. As a special case, though, such a component may be nearly identical for every condition and thus “condition-invariant.” That is, there might exist an *i^th^* component where $x_{i,c_{j},t}\approx x_{i,c_{k},t}$ for all choices of conditions $c_{j}$ and $c_{k}$ and times *t*. This more constrained possibility is suggested by a recent model (Sussillo et al., 2015) where the input that triggers movement generation produces population-level components that are close to condition-invariant.

The presence of a condition-invariant component versus a merely condition-correlated component can be determined only at the population level. To do so we applied dPCA (Machens et al., 2010; Brendel et al., 2011), a variant of PCA. Each component identified by dPCA is a pattern of responses across conditions and times (Eq. 1, $x_{i,:,:}$) from which the response of each neuron in the population is composed. dPCA exploits knowledge discarded by traditional PCA: the response of a neuron is not simply a vector of firing rates. Rather, each element of that vector is associated with a particular condition and time. dPCA attempts to find components that vary strongly with condition (but not time) or vary strongly with time (but not condition). In practice dPCA never found components of the first type; all components that varied with condition also varied with time. We term these components “condition-specific.” However, dPCA consistently found components that varied with time but not condition (i.e., that were condition-invariant).

**Figure 3.**
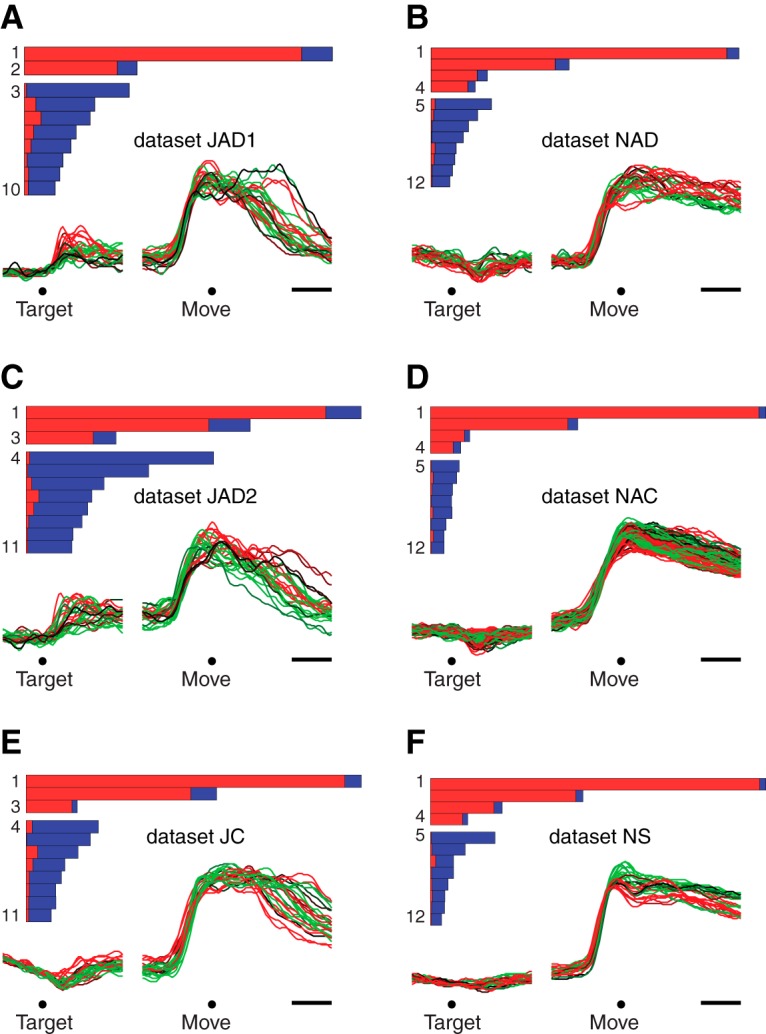
Performance of demixing on the empirical data. **A.** Bars show the relative variance captured by each dPCA component for dataset JAD1. Each bar’s horizontal extent indicates the total variance captured by that component. The red portion indicates condition-invariant variance, while the blue portion indicates condition-specific variance. Components are grouped according to whether they were overall condition-invariant (top group, >50% condition-invariant variance) or condition-specific (bottom group, >50% condition-specific variance). Traces show the projection onto the first dimension found by dPCA (CIS_1_) versus time. Each trace corresponds to one condition. *Target*, target onset; *Move*, movement onset. Scale bars, 200 ms. **B—F.** Same as A, for the remaining datasets.

Indeed, for every dataset the largest component found by dPCA was close to purely condition-invariant. Figure 3 quantifies the total variance captured by each component (length of each bar) and the proportion of that variance that was condition-invariant (red) versus condition-specific (blue). The largest component (top bar in each panel) exhibited 89–98% condition-invariant variance across datasets.

As a working definition, we term a component “condition-invariant” if >50% of the variance is condition-invariant. We term a component “condition-specific” if <50% of the variance is condition-invariant. Empirically components were either strongly condition-invariant (much greater than 50% condition-invariant variance) or strongly condition-specific (much less than 50% condition-invariant variance). Each bar plot in Figure 3 thus groups condition-invariant components at top and condition-specific components at bottom. All datasets contained multiple condition-invariant components: respectively two, three, three, four, four, and four for datasets JAD1, JAD2, JC, NAD, NAC, and NS. For a given dataset, we refer to the set of condition-invariant components as the CIS. We refer to the largest condition-invariant component as CIS_1_.

### Time course of the largest condition-invariant component

CIS_1_, like all the components, is a linear combination of individual-neuron responses; it is a “protoneural” response strongly reflected in single-neuron PSTHs. The structure of CIS_1_ can thus be plotted using the format typically used for a single-neuron PSTH. Figure 3 does so for each dataset (colored traces below bar plots).

CIS_1_ displayed a large and rapid change before movement onset that was similar across conditions. This pattern was present for all datasets. The sudden change occurred ∼150 ms before movement onset, corresponding to 50–100 ms before the first change in EMG activity (not shown). The condition-invariance of the signal can be visualized by noting that most individual traces (one per condition) overlap. In particular, during the moments before movement onset, CIS_1_ increases in a similar way and to a similar degree for every condition. Modest differences between conditions appeared primarily around the end of the movement and during the subsequent hold period (for reference, movement duration was on average 400 ms). Thus, while CIS_1_ was not identical across conditions, it was very close: on average 94% of its structure was dependent on time but not condition.

### The CIS is large

For every dataset, CIS_1_ captured the most variance of any single component. That is, CIS_1_ was the component that made the largest contribution to the response structure of individual neurons. More generally, the set of condition-invariant components (CIS; Fig. 3, top grouping of bars within each panel) together captured 49–77% of the total variance captured by dPCA (respectively 49, 49, 62, 67, 77, and 75% for datasets JAD1, JAD2, JC, NAD, NAC, and NS). Thus, not only is a CIS present, it typically comprises half or more of the data variance.

While each condition-specific component captured much less variance than CIS_1_, there were relatively more condition-specific components (Fig. 3, bottom groupings of bars) whose combined variance was 23–51% of the total variance captured by dPCA. These condition-specific components often contained preparatory activity followed by multiphasic responses during the movement. We return later to the structure captured by the condition-specific components.

We did not expect that such a large fraction of the structure in the data—half or more—would be condition-invariant. Most prior work (including our own) has concentrated on the tuned, condition-specific aspects of neural responses. This is reasonable: the presence of a large condition-invariant response component is not obvious at the single-neuron level. Essentially all neurons had contributions from condition-specific components and were therefore tuned for condition. Such tuning is the typical focus of analysis in most studies. Yet the fact that the CIS is so large argues that its properties should also be characterized.

While a few neurons (Fig. 2*C*,*D*) had an unusually large contribution from the condition-invariant components, we found no evidence for separate populations of condition-invariant and condition-specific neurons. Weights *w_n_*_,1_ were continuously distributed, and could be positive (Fig. 2*C*) or negative (Fig. 2*D*). We also note that the average $\left|w_{n,1}\right|$ was similar for neurons recorded in PMd and M1, indicating that the CIS is of similar size in the two areas.

### Assessing demixing

Importantly, dPCA cannot take condition-specific components and render them into condition-invariant components. This is true even if condition-specific components are strongly condition-correlated (see Materials and Methods for mathematical proof; empirical controls described below). Thus, the degree to which the population contains truly condition-invariant components can be assessed by the degree to which dPCA “demixes” responses; that is, the degree to which projecting onto orthogonal dimensions yields some response components that are close to purely condition-invariant. Demixing will be successful only if such condition-invariant structure is present in the data.

As noted above, demixing was successful for all datasets: most components were either strongly condition-invariant or strongly condition-specific. The condition-invariant components (Fig. 3, top grouping of bars in each panel) displayed 75–98% condition-invariant variance (mean, 88%). The condition-specific components (bottom grouping of bars) displayed 74–99% condition-specific variance (mean, 91%). As discussed above, the largest component—CIS_1_—was always very close to purely condition-invariant (mean, 94%). To put these findings in context, we analyze below a set of model and surrogate populations.

### A CIS in a network model

**Figure 4.**
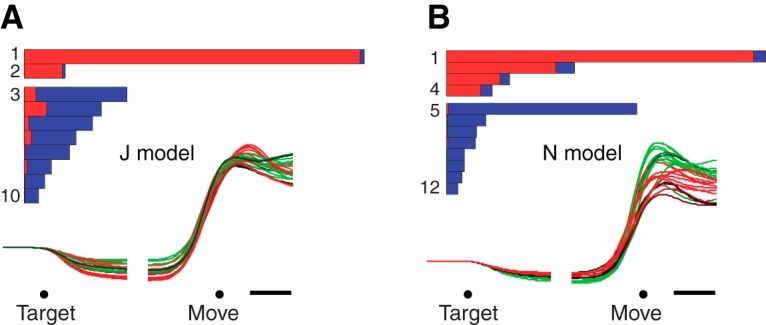
Same as Figure 3, but for the recurrent neural network models.

In addition to the six physiological datasets, we analyzed two model populations. The models were recurrent neural networks trained (Sussillo and Abbott, 2009; Martens and Sutskever, 2011) to generate the empirical patterns of muscle activity for two monkeys (Sussillo et al., 2015). Model populations exhibited a CIS (Fig. 4) that closely resembled that of the neural populations. In particular, there was a sudden change in CIS_1_ shortly before movement onset that was almost purely condition-invariant, with a small amount of condition-specific structure appearing after that transition. Similar to the neural datasets, CIS_1_ was the largest component of the data and was overall very close (99 and 96%) to purely condition-invariant. As with the physiological data, demixing was successful: the model population response could be separated into components that were either nearly condition-invariant (Fig. 4, top grouping of bars in each panel) or strongly condition-specific (bottom grouping of bars). The model datasets exhibited, respectively, two and four condition-invariant components—similar to the range of 2–4 seen for the empirical datasets.

As will be shown below, a CIS is not a general feature of any large complex dataset. In the case of the model, the presence of a strong CIS is a consequence of the network inputs (which include a condition-invariant trigger signal) and of the “strategy,” found via optimization, by which the network solves the task. The network was designed such that condition-specific preparatory inputs produce network states (one per condition) appropriate to seed subsequent movement-period dynamics. Those movement-period dynamics are “turned on” by a strong triggering input that contains no condition-specific information. Because the network was optimized to achieve smooth dynamics, nonlinear interactions are modest, and the triggering input produces a signal that is nearly condition-invariant in the population response. Whether the neural data exhibit a CIS for similar reasons remains unknown, but the temporal structure of the CIS is remarkably similar for the model and for the data.

### Controls: comparison of dPCA and PCA

One potential concern is that an algorithm such as dPCA might be able to “successfully” demix any high-dimensional data and find a condition-invariant component. As discussed above (and shown formally in the Materials and Methods), it is not in general mathematically possible to find a condition-invariant component if one is not truly present. Yet in practice, for a finite number of conditions, random smooth data will likely contain some (probably low-variance) signal that may be roughly condition-invariant. Is the empirical CIS larger than expected given this potential concern? Is the CIS found simply because dPCA attempts to find it?

One way to address this concern is to compare the performance of dPCA with that of PCA. PCA identifies dimensions that capture the most data variance possible. If dPCA achieved spurious demixing by finding components with the desired structure but little variance, then dPCA should capture much less variance than PCA. In fact, the dimensions found via dPCA captured almost as much variance as the dimensions found via PCA. Specifically, the set of dPCA dimensions captured 96–99% as much variance as the same number of PCA dimensions. Furthermore, the projections onto the first two PCA dimensions showed a structure that was naturally very close to condition-invariant. This was a simple consequence of the fact that the first few dimensions found by dPCA and PCA were very similar: the first dimension found via PCA formed an angle of only 5° on average with the first dimension found by dPCA. This was true for both the neural and model data. Thus, dPCA simply allows one to gain an ideal view of a condition-invariant structure that is naturally present in the data.

### Controls: demixing of real and surrogate data

Despite the above control, one might remain concerned that perhaps any generic data will tend to contain a condition-invariant component that would become apparent when applying dPCA (or PCA). A related potential concern is that a CIS might be found simply because firing rates are constrained to be positive. We addressed these potential concerns by applying dPCA to various surrogate datasets.

First, for each empirical dataset, we replaced each neuron’s response with a random set of responses that was matched with that neuron for frequency content (see Materials and Methods). Across 1000 repetitions for each of the six datasets, dPCA never identified a component with >18% condition-invariant variance. In contrast, the original data contained components with up to 98% condition-invariant variance. This control thus demonstrates that “random” data is very unlikely to yield a strongly condition-invariant component, even when temporal smoothness is matched to that of the empirical data. However, although the randomized responses (not shown) are frequency-matched to the data, they do not form realistic-looking PSTHs because the phases have been randomized (they are essentially just filtered noise). The second and third controls below, in contrast, do result in surrogate responses that look realistic at the level of PSTHs.

**Figure 5.**
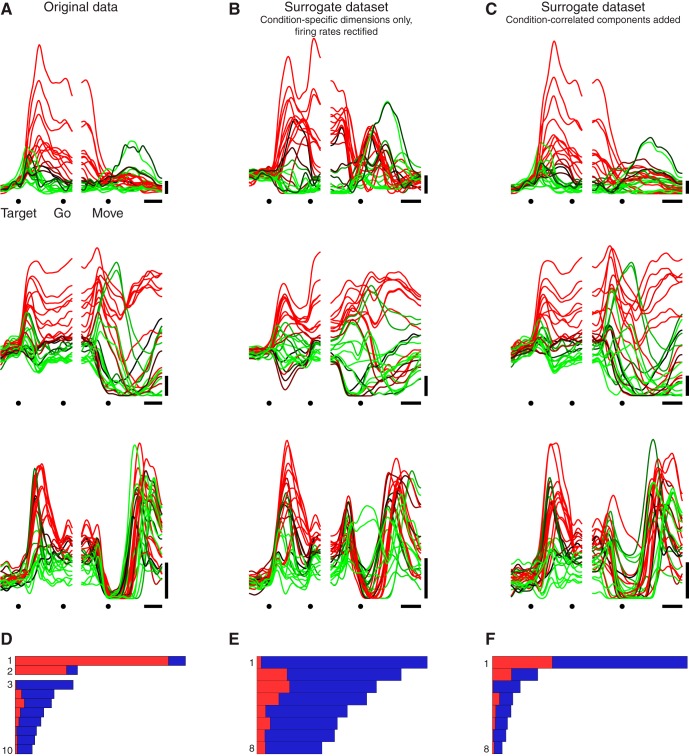
Demixing performance for one empirical dataset and two surrogate control datasets. ***A***, PSTHs of three example units from dataset JAD1. Scale bars: horizontal, 200 ms; vertical, 10 spikes/s. ***B***, PSTHs for a surrogate dataset where we projected onto the condition-specific dimensions, then rectified so that all firing rates remained positive (see Materials and Methods). This surrogate dataset explores the possibility that a CIS might appear merely due to firing rates being constrained to be positive. The three PSTHs correspond to the same units shown in ***A***, after modification. ***C***, PSTHs for a surrogate dataset where we added condition-correlated components. The condition-correlated components had the same temporal profile as the projections onto the condition-invariant dimensions found by dPCA but had a different amplitude for each condition (see Materials and Methods). This surrogate dataset explores whether a condition-correlated structure at the single-neuron level is sufficient to yield condition-invariant components at the population level. The three PSTHs correspond to the same units shown in ***A***, after modification. ***D–F***. Quantification of the CIS as in Figure 3. Panels correspond to examples above. ***D*** is reproduced from Figure 3*A* for comparison.

For the next control, we produced surrogate datasets by removing the CIS from each real neuron’s response and then applying a firing-rate threshold at zero (see Materials and Methods). The goal was to determine whether it was possible to produce an artifactual CIS by constraining firing rates to be positive. None of these surrogate populations exhibited a CIS. For example, for the original dataset JAD1, CIS_1_ contained >90% condition-invariant variance (Fig. 5*A*,*D*). The corresponding control dataset (Fig. 5*B*,*E*) had no CIS components; all components had <50% condition-invariant variance. For each of the six surrogate datasets, the first component found by dPCA had <21% condition-invariant structure (mean, 6%), in strong contrast to the data where the first component was always strongly condition-invariant. Thus, if a population response does not contain a CIS, a CIS is not created via the constraint that firing rates must be positive.

Finally, we wished to perform a control that could address both of the above concerns while preserving the surface-level features of the original data as closely as possible. To do so, we began with the original neural population (Fig. 5*A*) and added condition-correlated components (see Materials and Methods). These condition-correlated components had the same temporal profiles as the original condition-invariant components, but the response had a different magnitude for each condition. The surrogate population possessed single-neuron responses (Fig. 5*C*) that looked remarkably similar to the original responses, and exhibited changes in the average across-condition firing rate that were almost identical to the original responses. Yet the surrogate population lacked any CIS (Fig. 5*F*). There were no components with >50% condition-invariant variance for any of the surrogate populations, even though these are prominent in all the empirical datasets.

In summary, the presence of a CIS requires a very specific population-level structure and does not arise as a simple consequence of single-neuron response features. Of course, the presence of a CIS is fully consistent with prior work where fits to single-neuron firing rates (e.g., directional tuning curves) typically require a nondirectional component. However, a nondirectional component would also be required when fitting the surrogate responses in Figure 5*B*,*C*, which contain no CIS. Thus, the presence of a CIS is consistent with, but not implied by, prior results at the single-neuron level.

### Relationship of the CIS to reach speed and muscle activity

**Figure 6.**
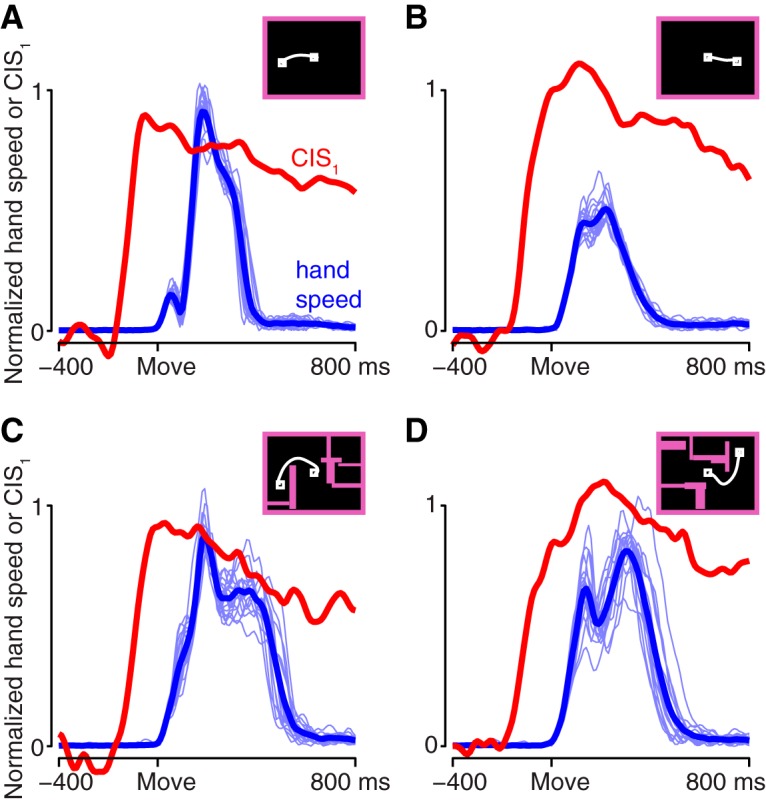
Comparison of the CIS and hand speed. Hand speed (blue) and the first component of the CIS (red) are shown for four reach conditions. For hand speed, light traces show all trials; heavy trace shows mean over trials. CIS_1_ is the mean over trials. Insets show the maze for that condition and a prototypical reach path. ***A***, A straight reach with a fast speed profile. Maze ID25. ***B***, A straight reach with a slow speed profile. Maze ID7. ***C***, A curved reach with a long speed profile. Maze ID5. ***D***, A curved reach with an unusual speed profile. Maze ID14. The CIS was similar across all four examples, despite differences in the speed profile. Dataset NAD.

For the model of Sussillo et al. (2015), the CIS plays an “internal role”: it reflects the arrival of a trigger signal that recruits strong dynamics. Might the CIS in the neural population play a similar internal role? Or might it be more readily explained in terms of external factors: for example, some aspect of kinematics or muscle activity that is invariant across conditions? In particular, tuning for reach speed has been a natural and reasonable way to model nondirectional aspects of single-neuron responses (Moran and Schwartz, 1999). However, for three reasons, the population-level CIS is unlikely to directly reflect reach speed. First, the CIS had a rather different profile from reach speed, which was more sharply phasic (lasting as little as ∼200 ms, depending on the condition) and returned to zero as the movement ended (Fig. 6, red trace and blue trace have very different temporal profiles). Second, for the task used here, reach speed is not condition-invariant: it varies considerably (∼2×) across the different distances and reach curvatures. Finally, even the small variations present in the CIS across conditions did not parallel variations in reach speed. For monkey J, peak speed and the peak magnitude of CIS_1_ were not significantly correlated (Fig. 6*A*,*B*; overall *r* = 0.097, *p* = 0.63 for JAD1; *r* = −0.018, *p* = 0.93 for JAD2). For monkey N, they were anticorrelated (*r* = −0.502, *p* = 0.008 for NAD, *r* = −0.364, *p* < 0.001 for NAC). Thus, the CIS and reach speed bore little consistent relation. As a side note, the dissimilarity between the CIS and hand speed does not imply that speed information could not be decoded. Using regression, we could identify a dimension that predicted speed fairly well (JAD1: *r* = 0.663; JAD2: *r* = 0.743; NAD: *r* = 0.833; NAC: *r* = 0.720), consistent with prior results that have found strong correlations between neural responses and reach speed (Moran and Schwartz, 1999). The projection onto this dimension, however, captured much less variance (4–16% as much) than CIS_1_.

**Figure 7.**
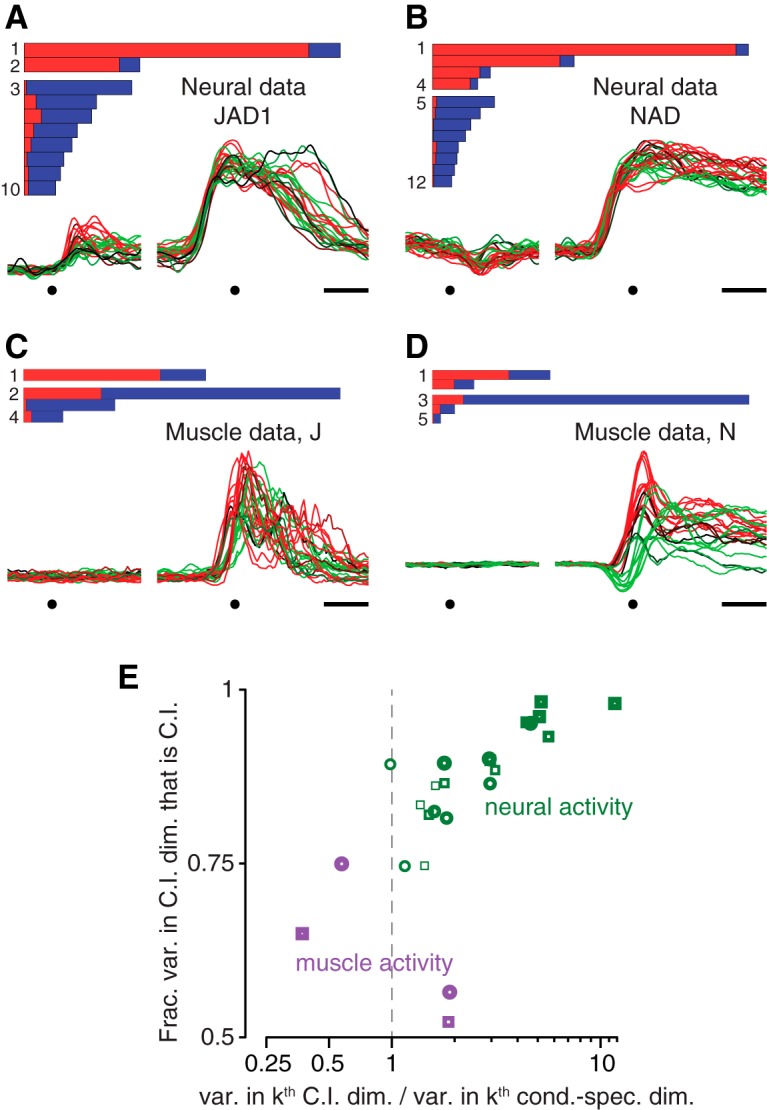
Comparison of dPCA applied to neural and muscle populations. ***A***, ***B***, Demixing performance (bars) and the projection onto the first dimension found by dPCA (CIS_1_) for neural datasets JAD1 and NAD. Each trace corresponds to one condition. These panels are reproduced from Figure 3*A*,*B* for comparison with the corresponding analysis of EMG. Dots indicate target onset and movement onset. Scale bar, 200 ms. ***C*, *D***, Similar analysis as in ***A*** and ***B***, but for the muscle populations recorded for monkeys J and N. Muscle activity was recorded for the same sets of conditions as for the neural data in ***A*** and ***B***. ***E***, To compare the prevalence of a condition-invariant structure in the neural and muscle populations, we focused on nominally “condition-invariant” components with >50% condition-invariant variance. There were many such components for the neural populations (green) and 1–2 such components for each of the muscle populations (purple). For each such component, two measurements were taken: the fraction of the component’s variance that was condition-invariant (vertical axis) and the total variance captured. The latter was expressed in normalized terms: the variance captured by the *k* ^th^ nominally “condition-invariant” component divided by the total variance captured by the *k* ^th^ “condition-specific” component (horizontal axis). Only the neural datasets contained components that were both strongly condition-invariant (high on the vertical axis) and that captured relatively large amounts of variance (to the right on the horizontal axis). Heaviest symbols correspond to the first dimension found by dPCA for each dataset; higher-numbered dimensions are plotted as progressively lighter symbols. Dashed gray line highlights variance ratio of unity. Circles, monkey J datasets; squares, monkey N datasets.

A related possibility is that the CIS might reflect nondirectional aspects of muscle activity. We performed dPCA on EMG recordings made from 9–11 key arm and shoulder muscles. The muscle populations did not exhibit a strong CIS. This can be seen by comparing the first component found via dPCA of the neural data (Fig. 7*A*,*B*) with the first component found via dPCA of the muscle data (Fig. 7*C*,*D*). The former is nearly condition-invariant while the latter is not. For each component found via dPCA, we measured the fraction of variance that was condition-invariant (the “purity” of condition-invariance) and the variance accounted for relative to the condition-specific components (the “strength” of that component). Unlike the neural populations (Fig. 7*E*, green) the muscle populations (purple) did not contain condition-invariant components that were both relatively pure and reasonably strong; there are no purple symbols in the upper right corner. Certainly the muscle population response contained some nondirectional aspects: there existed components in which there was an overall change that was mostly of the same sign across all conditions, resulting in a proportion of condition-invariant variance as high as 0.5–0.75 (purple symbols, left). This variance is not negligible, as evidenced by the fact that it could be further reduced via the control manipulations that were applied to the neural population in Figure 5 (muscle version not shown). However, the components in question captured relatively modest amounts of variance, and were not nearly as pure as the components found for the neural populations. Thus, the presence of condition-invariant structure in the neural population cannot be secondary to features of the muscle activity: only the neural population contained components that were both close to purely condition-invariant and captured a large percentage of the overall variance.

The muscle responses further underscore that the presence or absence of a CIS cannot be inferred from surface-level features. Individual muscle responses closely resembled neural responses in many ways, and often showed overall rises in activity across conditions. Thus, fits to muscle activity would benefit from a nondirectional component just as do fits to neural activity. Yet as a population, the muscles showed only condition-correlated structure, and had little or no CIS.

### Trial-by-trial prediction of RT

In all datasets, the sudden change in the CIS occurred ∼150 ms after the go cue and ∼150 ms before the onset of physical movement (50-100 ms before muscle activity began to change). The change in the CIS might thus be a visuospatial response locked to the go cue, consistent with the presence of other visuospatial signals in the premotor cortex (Crammond and Kalaska, 1994; Shen and Alexander, 1997). Alternatively, the change in the CIS could be locked to the transition from preparation to movement, consistent with the model of Sussillo et al. (2015). These two possibilities can be distinguished at the single-trial level. If the CIS reflects the visual go cue, it would have no ability to predict the subsequent variable RT between the go cue and movement onset. If the CIS reflects an internal transition related to movement onset, the CIS should be strongly predictive of RT.

We were able to address the trial-by-trial timing of the CIS in three datasets (JAD1, JAD2, and NAD) that were collected specifically for this purpose. These datasets involved simultaneous recordings (116–213 units) from two chronically implanted 96-electrode arrays, allowing single-trial estimates of the CIS. Critically, for these datasets we employed a task structure that allowed examination of trial-by-trial RT variability independent of delay-period duration. Over the course of training and most experiments, monkeys experienced a continuous distribution of delay-period durations from 0 to 1000 ms. It is well known that delay-period duration has an impact on RT (Rosenbaum, 1980; Riehle and Requin, 1989; Churchland et al., 2006a). To study RT variability independent of such effects, for these three datasets we interleaved additional trials with a discrete set of delay durations: 0, 100, 200, and 500 ms (see Materials and Methods). This allowed us to examine the relationship between neural and RT variability for sets of trials with a matched delay. Below we present data for trials with zero delay and trials with a “long” (500 ms) delay. Results were very similar when we analyzed the sets of trials with 100 and 200 ms delays. For comparison, we repeated these analyses of RT for dataset NAC (which did not contain discrete delays) using all trials with delays >150 ms. All results were very similar across all four datasets.

**Figure 8.**
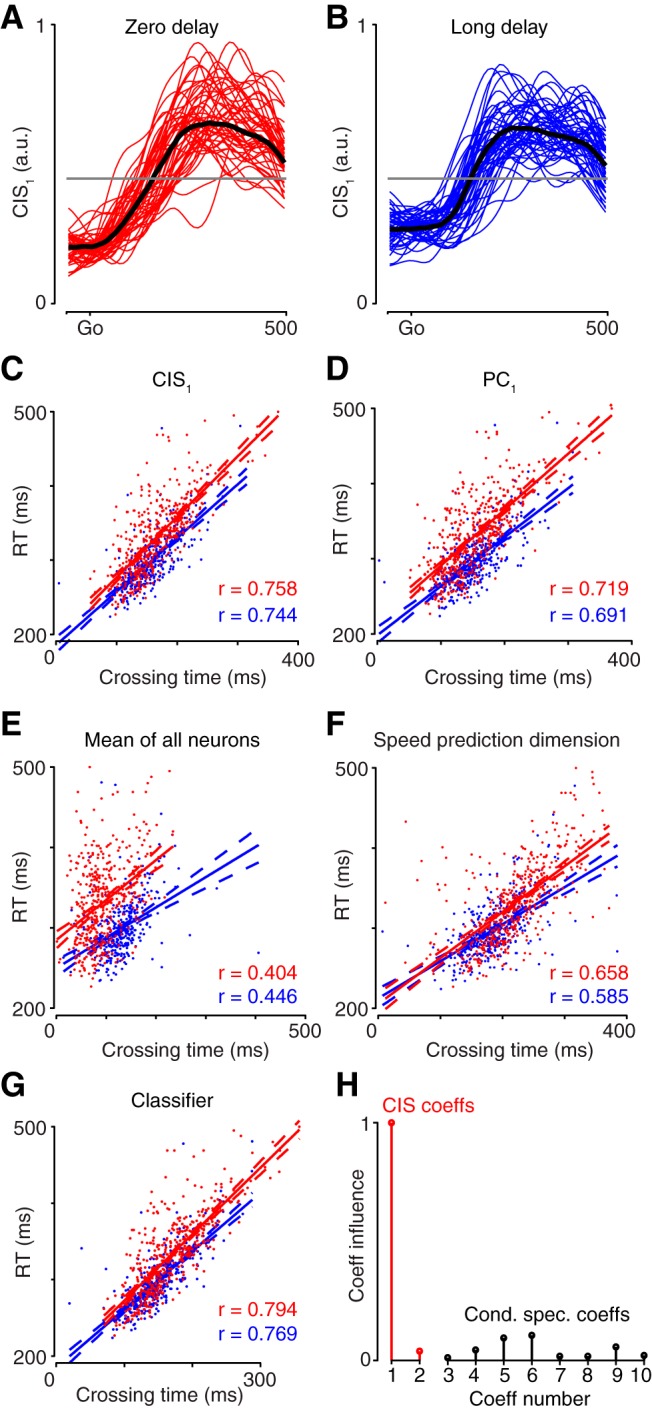
Predicting RT using projections of the data for dataset JAD1. ***A***, Each trace plots CIS_1_ over time on a single zero-delay trial. Fifty trials selected randomly at intervals throughout the day are shown. Black trace plots the median across all trials. ***B***, Same as in ***A*** but for trials with a 500 ms delay period (long delay). The criterion value (gray line) was chosen using long-delay trials. The same value was used for zero-delay trials (***A***). ***C–G***, Correlation of behavioral RT with the time when the neural criterion value was crossed. For each panel, data are shown for both long-delay trials (blue) and zero-delay trials (red). Lines show linear regressions; dashed lines show 95% confidence bounds of the fit. Each panel in ***C–G*** gives the correlation for a different linear projection of the population response: CIS_1_ (***C***), the projection onto the first PC (***D***), the mean over all neurons (***E***), the projection onto the dimension that best reconstructed speed according to a linear regression (***F***), and the projection onto the axis found by a logistic regression classifier (***G***). Trials where the neural data did not cross the criterion value were excluded. ***H***, Coefficient influence for the classifier. Coefficients for condition-invariant dimensions shown in red; condition-specific dimensions shown in black.

**Figure 9.**
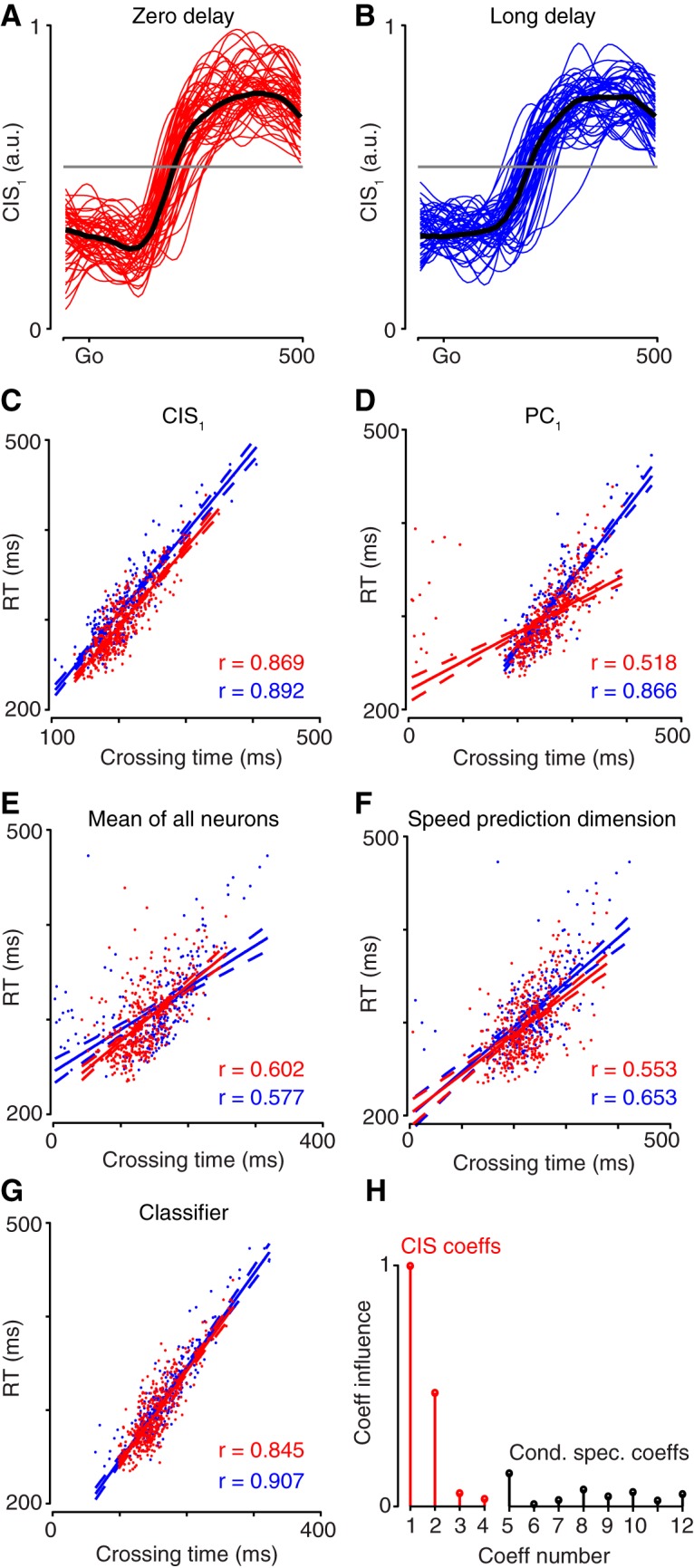
Predicting RT using projections of the data for dataset NAD. Same format as Figure 8. Regarding ***H***, note that for this dataset there were four CIS components.

CIS_1_ was readily resolved on individual trials (Fig. 8 shows data for JAD1 with analyses repeated in Fig. 9 for NAD). The neural weights defining CIS_1_ were found using data from the long-delay trials. Example single-trial projections of the long-delay data are shown in Figures 8*B*and 9*B*. These same weights successfully generalized and revealed an essentially identical CIS_1_ for the zero-delay trials (Figs. 8*A*, 9*A*). The latency of the rise time of CIS_1_, relative to the go cue, varied from trial to trial. To estimate this latency, we measured when CIS_1_ crossed a criterion value following the go cue (Figs. 8*A*,*B*, 9*A*,*B*, gray line). We selected a 50% criterion that is simply a practical and robust criterion for estimating rise time (and should not be interpreted as suggesting a physiological threshold). The estimated rise time strongly predicted the subsequent RT on individual trials (Figs. 8*C*, 9*C*) for both long-delay (blue) and zero-delay (red) trials. This was true across all analyzed datasets: the average correlation was *r* = 0.805 for long-delay trials, and *r* = 0.827 for zero-delay trials.

The CIS strongly predicts RT on a single-trial basis, but does it do so more accurately than other reasonable methods? The projection of the data onto the first principal component of the data (PC_1_) predicted RT almost as well as did CIS_1_. This was especially true for monkey J (Fig. 8*D*) and somewhat less so for monkey N (Fig. 9*D*) due to a tendency for the projection onto PC_1_ to occasionally exceed the criterion early. Given the ability of CIS_1_ to predict RT, the similar success of the projection onto PC_1_ is unsurprising: as discussed above, the dimensions containing PC_1_ and CIS_1_ were closely aligned. Nonetheless, CIS_1_ always predicted RT at least slightly better than the projection onto PC_1_, despite PC_1_ capturing (by construction) slightly more variance. The average firing rate across all neurons (Figs. 8*E*, 9*E*) predicted RT less well than did CIS_1_ or the projection onto PC_1_. Finally, because RT was quantified based on measured hand speed, we considered the projection that best decoded hand speed (found via regression; see above). Decoded hand speed performed acceptably, but noticeably less well than CIS_1_ (Figs. 8*F*, 9*F*; across all analyzed datasets, mean *r* = 0.666 for long delay, *r* = 0.674 for zero delay). Thus, CIS_1_ predicted RT better than did other reasonable unsupervised and semisupervised methods.

Might there exist another signal in the data that could considerably outperform CIS_1_? To address this, we trained a classifier based on logistic regression (see Materials and Methods) to distinguish neural data recorded before versus after the sudden transition in neural activity 150 ms before movement onset. The classifier—which has the advantage of being optimized using knowledge of RT—predicted RT for zero-delay trials slightly better than CIS_1_ for one dataset (Fig. 8*G*) and slightly worse for the other (Fig. 9*G*; note that when assessing generalization, a supervised method is not guaranteed to outperform an unsupervised method). We then asked which dimensions the classifier relied upon. The coefficients of the classifier (Figs. 8*H*, 9*H*) revealed that the condition-invariant dimensions (red) were used more strongly than the condition-specific dimensions (black); 74% of the classifier was based on the CIS (79% for dataset NAD). Thus, the CIS is a particularly good predictor of RT, and it is difficult to improve on the performance it provides. Results were similar for the other two datasets (for dataset JAD2: 69% of classifier based on CIS; dataset NAC: 82% of classifier based on CIS). Thus, the timing of the CIS reflects the pending onset of movement, rather than the arrival of a visual signal. Had the latter been true, the CIS would have had no ability to predict RT when data are time-locked to the go cue as they were here.

### Neural and model population trajectories

**Figure 10.**
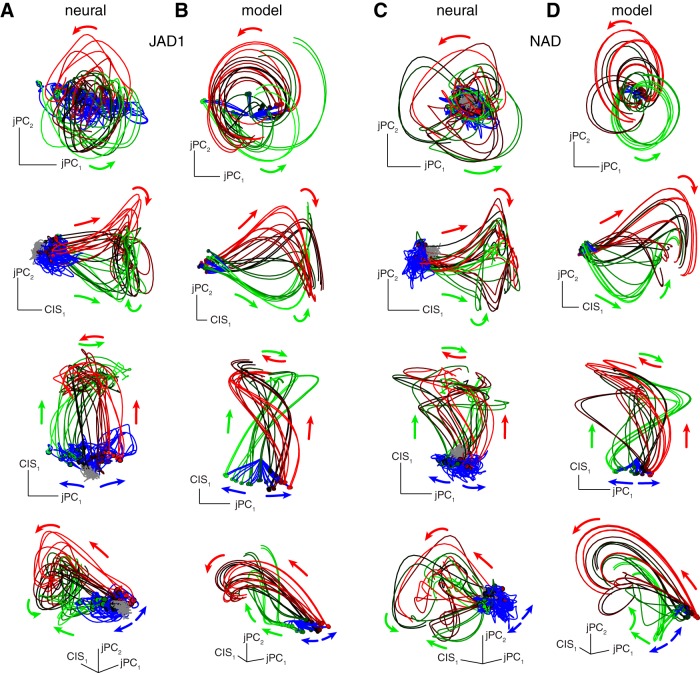
Various projections that capture CIS_1_ and rotations of the neural state during movement. Data were first projected onto three dimensions: the dimension that yielded CIS_1_ and two condition-specific dimensions that captured strong rotational structure (see Materials and Method*s*). Each panel plots a different view of the data projected onto those three dimensions. ***A***, Four different views of the three-dimensional projection for dataset JAD1. “Baseline” activity (before target onset) plotted in gray, preparatory activity plotted in blue, and activity after the go cue plotted in green and red (colors chosen arbitrarily for each condition). ***B***, Same for the neural network model trained to produce EMG recorded from monkey J. ***C***, Same for dataset NAD. ***D***, Same for the neural network model trained to produce EMG recorded from monkey N.

We recently reported that the population response exhibits a strong ∼2 Hz oscillatory component during movement, manifested as a rotation of the neural state (Churchland et al., 2012; Churchland and Cunningham, 2014). This oscillatory component is condition-specific: rotation amplitude and phase differ across reach directions, curvatures, speeds, and distances. As expected given these prior results, we found that the eight-dimensional condition-specific space identified via dPCA contained components with a strong rotational structure. This conveniently allows the population structure to be plotted as a neural trajectory in a state space, with one dimension capturing CIS_1_ and two dimensions capturing the plane with the strongest rotations. The resulting three-dimensional projections captured 47 and 45% (for datasets JAD1 and NAD respectively) of the total variance captured by dPCA. The three-dimensional structure is best viewed in video format (Movies 1–4) but can also be appreciated via inspection of a set of two-dimensional projections (Fig. 10*A*,*C*).

Movie 1. Three-dimensional view of Figure 10*A* (dataset JAD1), rotating to display structure. Axis labeled CIS corresponds to CIS_1_. 10.1523/ENEURO.0085-16.2016.video.1

Movie 2. Three-dimensional view of Figure 10*C* (dataset NAD), rotating to display structure. 10.1523/ENEURO.0085-16.2016.video.2

Movie 3. Three-dimensional view of Figure 10*A*, bottom (dataset JAD1), with events unfolding over time. Movie starts 300 ms before target onset, and ends 400 ms after movement onset. 10.1523/ENEURO.0085-16.2016.video.3

Movie 4. Three-dimensional view of Figure 10*C*, bottom (dataset NAD), with events unfolding over time. Movie starts 300 ms before target onset, and ends 600 ms after movement onset. 10.1523/ENEURO.0085-16.2016.video.4

Each trace in Figure 10 and Movies 1–4 plots the neural trajectory for one condition. Traces are colored gray during baseline, blue during the delay period, then shaded from red to green across conditions (to aid visualization) during a “perimovement period”: −200 to +150 ms relative to movement onset. The overall structure carved out by the trajectories is roughly conical; neural activity is at the narrow end of the cone during the delay period, translates along the long axis of the cone just before movement onset, then exhibits rotations at the wide end of the cone during movement. Rotations begin with (or just at the end of) the translation and continue after the translation is over, tracing out a rough disk. The top row plots projections in which the cone is seen end-on. Middle rows plot projections in which the cone is seen from the side (the rotational disk being viewed from the edge) and the bottom row plots a projection that illustrates (as best as possible in two dimensions) the full three-dimensional structure.

Consistent with many reports demonstrating the existence of preparatory activity (Tanji and Evarts, 1976; Weinrich and Wise, 1982; Hocherman and Wise, 1991; Messier and Kalaska, 2000; Churchland et al., 2006b), condition specificity first develops during the delay period. For example, in the third row, blue traces spread out over a larger range of states than do gray traces. The subsequent rotations are also condition-specific. The CIS produces the long axis of the cone: a large translation of the neural state that is similar for every condition. This translation is almost perfectly orthogonal to the rotations. Such orthogonality is not a consequence of the analysis method: the axes are orthogonal by construction, but that in no way constrains the condition-invariant and condition-specific structure to be orthogonal. Indeed, demixing (Fig. 3) is successful precisely because the condition-invariant and condition-specific response structure is orthogonal, as revealed directly in Figure 10. The other four datasets showed the same structure.

A striking feature of the response structure is that condition-specific preparatory activity occurs in one region of state space, while condition-specific rotational structure during movement occurs in a different region of state space. Given the above results showing that the CIS predicts RT, a natural question is whether the transition from one region to another relates to the behavioral transition from preparing to move (while holding a steady posture) to actually moving. This is indeed how the network model of Sussillo et al. (2015) functions. Through optimization, that model adopted a strategy where an incoming “trigger signal” produced a large translation, bringing the population state near a fixed point where local dynamics were rotational and produced the multiphasic patterns of muscle activity. That study noted the general similarity between neural and model data, as revealed via canonical correlation analysis, and the presence of a change in the overall mean firing rate. That overall change is a natural product of the CIS, which as documented above is present in both neural (Fig. 3) and model (Fig. 4) populations.

To further compare, we projected the model population response (Fig. 10*B*,*D*) as we had the neural population response. Model and neural populations exhibited remarkably similar structure when viewed from all angles. Preparatory activity developed in one region of space, and the CIS then caused an overall translation to another region of space. The rotations of the neural state (at a little less than 2 Hz) began during that translation and continued to unfold after the translation was complete.

### Relative timing of the CIS and rotations

**Figure 11.**
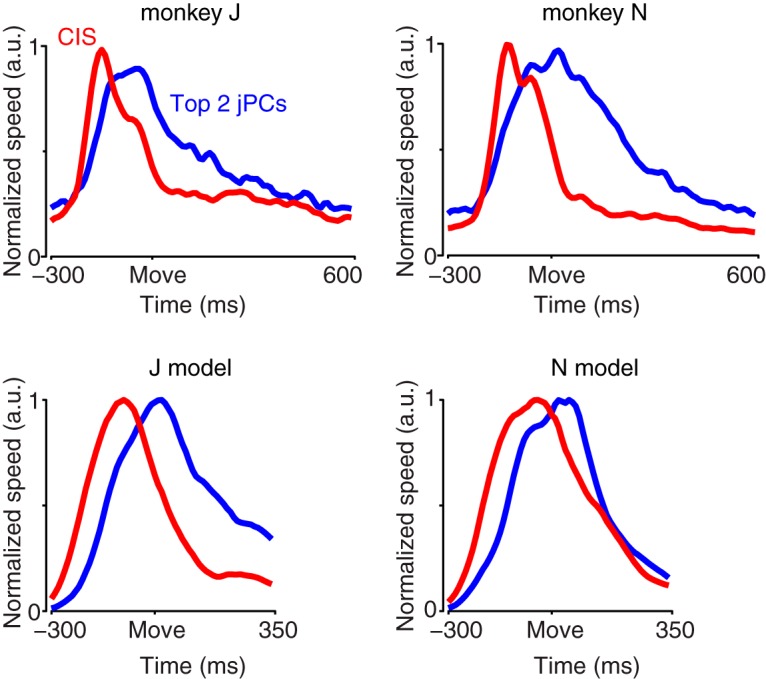
Comparison of the temporal profile of the trajectory of the CIS and the temporal profile of the condition-specific rotational patterns. The vertical axis plots “neural speed”: the rate of change of the neural state in the condition-invariant dimensions (red) and in the first jPCA plane (blue), which captures the strongest rotations. The rate of change was computed separately for each condition, then averaged across conditions. For each dataset that average was normalized by its maximum. For statistical power, results for the neural data were averaged across the three datasets for each monkey. Move, Movement onset. Note that because the data have been smoothed and differentiated, the first moment when the state begins to change is shifted leftwards: the CIS appears to begin changing >200 ms before movement onset, when ∼150 ms is a more accurate estimate (Fig. 3). Since both the condition-invariant dimensions and the jPCA dimensions are processed in the same way, however, their relative timing can be compared.

The above results suggest that the CIS may relate to the transition from relatively stable preparatory dynamics to strongly rotational movement-period dynamics. This hypothesis makes a specific prediction: the CIS should begin to change with, or perhaps shortly before, the onset of rotational dynamics. The hypothesis would be falsified if the CIS began changing after rotations had already begun, or if the CIS began changing long before rotations began. To assess relative timing, we computed the “speed” of the neural trajectory: the rate of change of the neural state. This was done separately for the CIS dimensions and the two dimensions with the strongest rotations (Fig. 11). In all cases, for both the model and data, the peak speed in the CIS dimensions (red) slightly leads the peak speed in the rotational dimensions (blue). Thus, both the neural and model data showed the predicted effect.

## Discussion

We found that the largest component of the population response in M1/PMd is consistently condition-invariant: it changes in an almost identical fashion regardless of reach direction, curvature, and distance. More generally, a small set of condition-invariant components (the CIS) contained half or more of the population-level variance. Thus, although essentially all individual motor cortex neurons are “tuned,” the population response is dominated by the CIS. This result could not be inferred from, but is consistent with, three prior findings. First, single neurons often exhibit an overall change in firing rate during movement (e.g., with most conditions showing an increase in rate, or most conditions showing a decrease in rate; Fortier et al., 1993; Crammond and Kalaska, 2000). Second, a strong nondirectional ensemble response is present in motor cortex (Moran and Schwartz, 1999; Churchland and Shenoy, 2007) such that fits are greatly aided by a nondirectional term (Georgopoulos et al., 1986; Moran and Schwartz, 1999). Third, population summaries often show a rise in activity for both the “preferred” and “antipreferred” direction around the time of the movement (Bastian et al., 2003). Yet importantly, the presence of the CIS could not be directly inferred from the above findings; they are all equally consistent with structure that is condition-correlated but far from condition-invariant. For example, the surrogate data in Figure 5 show all three of the above features yet lack any condition-invariant component. In summary, the current data and analyses reveal something that could not be inferred previously: the data contain condition-invariant components that constitute a very large percentage of the overall structure of the neural responses.

### Temporal properties of the CIS

Although one might initially be tempted to view untuned response aspects as “nonspecific,” the CIS exhibits specific temporal structure. For all six neural datasets and both model datasets, there is a sudden change in the CIS ∼150 ms before movement begins. The sudden change can be visualized on individual trials and is strongly predictive of trial-by-trial RT. This strong relationship reflects the fact that the CIS is tied to movement onset (rather than the appearance of the go cue) and is large enough to be readily measured on single trials. The CIS also has a specific population-level structure that was consistent across datasets: the CIS is manifested as a large translation of the neural state from one region of neural state space (occupied when the monkey is preparing the movement) to another region (occupied just before and during overt movement).

While neural responses are often interpreted in terms of their tuning for external factors, the CIS did not relate to any external factor we examined. The temporal profile of the CIS did not resemble that of hand speed, nor were condition-to-condition variations in hand speed paralleled by the (very small) condition-to-condition variations in the CIS. This is consistent with the noisiness associated with decoding pure hand speed in neural prosthetics (Golub et al., 2014), and suggests that the CIS could be useful for applications seeking to decode a rest versus a move signal (Velliste et al., 2014). The CIS also did not relate to any measureable aspect of muscle activity. Although muscles often exhibited overall changes in activity that were correlated across conditions, the muscle population exhibited little to no CIS. This again underscores that condition-correlated structure typically does not imply a CIS.

Finally, the CIS did not simply reflect the visual arrival of the go cue. As indicated by the ability to predict RT, the CIS was instead related to the time of movement onset. Furthermore, the sudden change in the CIS occurred well after (∼150 ms) the visual go cue. This contrasts with the very rapid (∼60 ms latency) response of neurons in M1 and PMd to the onset of the target (Ames et al., 2014; Fig. 2*A*,*B*,*D*). We also note that the visual go cue was far from condition-invariant: it involved salient changes in the appearance of the target(s), which had different visual locations across conditions. Thus, a natural interpretation is that the CIS relates to the go cue only indirectly, and reflects an internal transition from preparation to movement that follows the go cue with a long and variable latency. Still, we cannot rule out that the CIS is a long- and variable-latency visual response to the go cue, and that the reaction time inherits this variability. Addressing this possibility will require future experiments in which there is no sensory go cue.

Future experiments will also be required to address whether the timing of the CIS relates in any way to the last moment when movement can be suppressed. A recent hypothesis is that RTs are artificially long not because motor preparation is slow, but because “triggering” is conservative (Haith et al., 2016), leaving time for the movement to be altered or suppressed (Riehle et al., 2006; Scangos and Stuphorn, 2010; Mirabella et al., 2011). The relatively long ∼150 ms time between the go cue and the sudden change in the CIS, relative to the ∼60 ms latency of the first “preparatory” response, is consistent with this hypothesis.

### An internal role for the CIS?

The properties of the CIS suggest that it likely relates not to a representation of external factors, but to some internal process—perhaps the transition from preparatory neural dynamics to movement-related neural dynamics. It is becoming increasingly appreciated that many motor cortex signals may not relate cleanly to external parameters, and are more naturally explained in terms of their internal roles in computation (Reimer and Hatsopoulos, 2009; Chase and Schwartz, 2011; Shenoy et al., 2013; Churchland and Cunningham, 2014). The hypothesis that the CIS might relate to the transition from preparation to movement is further suggested by the finding that the network model of Sussillo et al. (2015) exhibits a very similar CIS—and similar overall population structure—to the neural data (Figs. 4, 10). In the case of the model, the CIS is a consequence of the externally delivered trigger signal, and is in turn the cause of the change in neural dynamics that generates movement. The original analyses in Sussillo et al. did not focus on or attempt to isolate a CIS. Yet a condition-invariant translation is clearly present in one key analysis (Sussillo et al., 2015, their Fig. 6) and can be seen to bring the set of network states close to a fixed point with rotational dynamics. Whether this interpretation is also correct for the data is of course still uncertain, but the population response structure is remarkably similar for the model and data. This interpretation is also supported by both the overall timing of the CIS (it occurs just as, or even slightly before, the onset of rotational dynamics; Fig. 11) and the remarkably strong correlation between the change in the CIS and the moment when movement begins (Figs. 8, 9).

Other, not necessarily exclusive explanations are also likely. For example, the CIS could activate, suppress, or alter how the local circuit processes feedback (Cluff et al., 2015). Similarly, the CIS could relate to an overall modulation of downstream reflexes or to a disengagement of postural control (Kurtzer et al., 2005; Cluff and Scott, 2016). After all, the initiation of activity that drives movement must presumably be accompanied by cessation of the activity that held the hand in place during the delay period. This is true even of the model of Sussillo et al., which is involved in a rudimentary form of postural control during the delay period: producing a constant pattern of muscle activity. For that model, the CIS produces the transition away from the stable dynamics that maintain constant outputs, and towards oscillatory dynamics that produce the movement-driving patterns of muscle activity.

### What inputs might produce a CIS?

If motor cortex undergoes a large condition-invariant change prior to movement, what drives that change? What other area(s) might supply the relevant input? A number of candidate regions exist, including the basal ganglia (Romo et al., 1992; Hauber, 1998), superior colliculus (Werner, 1993; Philipp and Hoffmann, 2014), parietal cortex (Scherberger and Andersen, 2007; Pesaran et al., 2008), supplementary motor area (Orgogozo and Larsen, 1979; Eccles, 1982; Romo and Schultz, 1992), the dentate nucleus of the cerebellum (Meyer-Lohmann et al., 1977), and, in rodents, secondary motor cortex (Murakami et al., 2014). Moreover, the origin of the CIS may depend on the task: movements elicited by a strong sensory cue may be generated differently from self-initiated movements (Kurata and Wise, 1988) or movements that must be made very rapidly (Perfiliev et al., 2010). Along similar lines, RTs can be remarkably short when the go cue is provided by a mechanical perturbation of the limb (Evarts and Tanji, 1976; Pruszynski et al., 2008). These short RTs may be related to the finding that some neurons show a rapid perturbation-driven response that is invariant across perturbation directions (Herter et al., 2009, their Fig. 3*C*). It is thus vital that future studies address whether a similar CIS is present in motor cortex across the many possible sensory cues and internal events that can be responsible for causing movement initiation.

### Summary

In summary, our results build upon the long-standing observation that responses are often correlated across conditions at the single-neuron level. Our results reveal that this general surface-level structure reflects a very particular kind of underlying structure: a large condition-invariant response component with timing closely tied to movement onset. This adds to a small but growing list of “untuned” response aspects that might initially appear incidental, but may in fact play important computational roles.

## Synthesis

The decision was a result of the Reviewing Editor Julie Andersen and the peer reviewers coming together and discussing their recommendations until a consensus was reached. A fact-based synthesis statement explaining their decision and outlining what is needed to prepare a revision is listed below. The following reviewers agreed to reveal their identity: Adrian Haith, Stephen Scott

The reviewers both felt that overall the data presented represents a significant advance in the field (clear evidence of motor action independent of precise planning), but have suggestions for improvements for revision and resubmission. The consensus on the part of the reviewers and the Reviewing Editor were that while the experiments were carefully planned and performed, there were issues with presentation (e.g. descriptions of the purpose of particular particular experimental paradigms, ambiguous usage of terms throughout the manuscript, lack of clarity as to how data was analyzed or what the outcomes really mean) to which specific recommendations have been included to help clarify precise meaning of the findings. This is particularly important for a broad journal like eNeuro which is read by neuroscientists in various sub-speciality areas. To this end, specific comments by both reviewers have been included below for consideration as part of your response to the review of your manuscript.

Reviewer #1:

The authors demonstrate the existence of a component of neural activity that contains little or no information about what movement is about to be performed, but contains very precise information about when a movement will occur. It has previously been suggested by many authors that movement initiation might be triggered independently of the processes of selecting or preparing an action. Here, the authors have identified clear evidence at the neural level for such a non-specific triggering mechanism. This distributed response within motor cortex might be driven by a simpler signal that originates from outside motor cortex.

This is an important contribution to our understanding of how movements are prepared and generated by the brain. The numerous results, control analyses etc are well thought through and I find the overall argument quite compelling. I did, however, find that the details of the analysis methods, and how exactly how the CIS is obtained, were unduly difficult to fully comprehend. After getting past the approach of how the CIS is established, however, the paper is generally a pleasure to read and I have no concerns about the actual scientific content, just about how some aspects of it are presented. I have outlined below a number of specific points of confusion/inconsistency which hampered my understanding at first, and which I hope can help the authors improve the overall exposition and comprehensibility of this otherwise excellent paper.

Signal/Component/Dimension/Pattern/Response:

These terms are used rather loosely and interchangeably throughout the manuscript. Sometimes many different words refer to the same thing, making it hard to be sure whether or not something new is being described. More troublingly, sometimes the same word is used to describe qualitatively different things. The main confusion is between whether these terms refers to a dimension in neural space, or to a time-varying profile of activity (this is particularly true for signal/response). In particular, the CIS seems to be introduced as a dimension or 'component' in neural space, but then transitions seamlessly into a time-varying 'signal' within this dimension. In general, I found that there was little clarity to when these two different senses were being used, and this ambiguity permeates the entire paper. More precise and consistent usage of these kinds of terms would greatly improve the clarity of the paper.

I note here a few illustrative examples where this ambiguity causes confusion, but in general this type of imprecise usage occurs throughout the paper.

line 99: "... the condition-independent signal underwent a large and sudden change ∼150 ms before movement onset and was an excellent predictor of reaction time." Is it the large and sudden change that is an excellent predictor of reaction time, or the condition-independent signal? If the latter (as the sentence is written), then 'signal' must be something inherently time-varying.

line 102: "... the condition-independent signal was almost perfectly orthogonal to the condition-specific components." If 'signal' is a time-varying quantity, then it's ambiguous how it can be 'orthogonal' to condition-specific components. Really it is the dimension in which this signal occurs that is orthogonal to the condition-specific dimensions.

line 586 "CIS_1, like all the components, is a linear combination of individual-neuron responses; it is a proto-neural response that is strongly reflected in single-neuron PSTHs". I think this statement is only meaningful if CIS_1 is a time-varying signal, rather than a direction in neural space. This is incompatible with the initial definition of CIS_1 as the output of dPCA - a dimension in neural space.

Other terminology:

Various points in the paper carefully describe how 'condition-specific' and 'condition-independent' are not necessarily dichotomous because of the possibility of 'condition-correlated' responses that are not in fact 'condition-independent'. Line 295 (and much of the results), however, introduces a completely different definition of 'condition-specific' meaning, essentially (if I have understood correctly), 'condition-specific or condition-correlated but not condition-independent'. It is of course, deeply confusing to offer two contradictory definitions of what it means to call something 'condition-specific' - and undermines a lot of the subtleties that the reader must navigate in order to appreciate the result.

The term "condition-correlated" is extremely confusing when used alongside the term "condition-independent". 'Condition-correlated' means correlated ACROSS conditions, while 'condition-independent' means independent OF condition. They are quite similar properties. In fact 'condition-independent' is a special case of 'condition-correlated'. Yet 'independent' and 'correlated' by themselves are opposites of one another. This incongruity will likely cause significant confusion to many or even most readers. One could imagine replacing 'condition-independent' with 'condition-identical', which is a much more natural counterpart to 'condition-correlated'. This would make the subtle arguments in the methods sections far easier to follow. But of course 'condition-identical' is not as nice a phrase as 'condition-independent', so coming up with an alternative to 'condition-correlated' would probably be preferable.

Description of dPCA:

Not having been previously been familiar with dPCA, I got very little insight into it's purpose or approach from reading this paper. Only after reading the original Brendel paper did I understanding the purpose of dPCA and, consequently, the results here. On line 560 dPCA is described as "a variant of PCA that can disentangle components that are mixed at the single-neuron level if they are segregated at the population level." This misses the key distinction of dPCA - that it utilizes additional labels usually discarded by PCA (condition, time) to help steer the dimensionality reduction in such a way that different dimensions segregate variability associated with these different labels. Without appreciating this use of additional labels, the notion of components being 'segregated at the population level' is quite meaningless.

line 267-269 couches dPCA as trying to segregate 'condition-specific' components from 'condition-independent' components. I feel that this slightly mischaracterizes the dPCA approach. In reality dPCA demixes 'time' and 'condition', not 'condition-specific' and 'condition-independent' - these are just properties that can be assessed after a subspace is identified. In general, the description of dPCA is heavily skewed towards the anticipated result. What is lost (or at least deeply buried) is the fact that dPCA actually treats 'time' and 'condition' symmetrically. It would actually be equally valid (and I would say equally unhelpful) to say that 'dPCA attempted to segregate time-specific components from time-independent components'. At present the critical importance of time in the analysis is mentioned only in passing, as something of an afterthought or caveat (e.g. line 261, 270), rather than as the primary idea driving the analysis. I suggest that it would be more transparent, informative and accurate to more clearly emphasize the 'time' vs 'condition' dichotomy that is the heart of the analysis, and present condition-independence as a potential property of the time-associated component extracted by dPCA. Rather than couching dPCA as a tool for identifying condition-independent components, while having to add the caveat that it might end up failing in subtle ways (like finding condition-correlated components).

An added benefit of putting 'time' and 'condition' on a more symmetric footing when describing the analysis is that it also affords some better intuition as to how a dimension can be condition-correlated but not condition-independent. Activity in the condition-dependent dimensions will of course also depend on time; it would be time-correlated but not time-independent. From here it is not too difficult to envisage how activity in the time-dependent dimensions might, in principle, also depend on condition in a similar manner.

Additional comments:

Why not plot the frequency-matched surrogate data in Figure 5? It would be very interesting and informative to see what these surrogate responses look like.

line 102 - "The condition-independent signal was almost perfectly orthogonal to the condition-specific components". I am struggling to understand how this is not inevitable given the dPCA approach. line 264 even states that "dPCA produces orthogonal dimensions..."

line 16: "...movement onset, the onset of which..." not the best wording... suggest rephrasing this sentence.

line 31 - not sure that I would agree that these signals are 'separate'. Separable, perhaps?

Reviewer #2:

The manuscript highlights that motor cortex displays a substantial amount of information reflecting the general act of reaching that is independent of the specific details of where the subject is reaching.

The present article explores how neural activity transitions from motor preparation to movement. Several complementary approaches highlight the presence of a signal related to the initiation of movement. The description of the analyses and results are generally quite thorough. The fact that multiple approaches find the same general conclusion is helpful to ensure the results are not likely due to details with regards to the mathematical techniques used to analyze the data. In general, I think this is a very novel observation. I have a few issues that should be considered.

The focus of thought in this manuscript is that neural processing in motor cortex is either related to movement preparation or movement execution. Missing from this is the fact that the monkey is having to perform an ongoing task, postural control at the start target. Admittedly, deep in the back of the discussion, the authors do get to the point that some of this condition-independent process may be related to disengagement of postural control. However, I think they should more fully recognize the intimate link disengagement of one motor act and initiation of another right from the beginning.

Omnidirectional responses are actually quite common following mechanical disturbances particularly neurons that respond rapidly after the disturbance (See Fig. 3 in Herter et al., JNP 2009). Mechanical disturbances also have a unique ability to dramatically speed up reaction times. It is commonly known from many human studies that large perturbation responses hasten simple reaction times from ∼120ms to about 60ms, commonly termed triggered reaction. It may be that this strong omnidirectional signal plays a key role in hastening reaction times whether initiated by a visual or a proprioceptive input to the network.

Figure 3 highlights that the first dPC (CIS1) has a temporal profile that extends well past movement onset. Thus, the condition-independent components may also reflect a generalized control policy for reaching that is present whenever reaching is performed. Added to this general reaching control policy is target dependent information reflecting specific aspects of the reach being performed.

Line 191: I think it is a bit dangerous to analyze multi-unit signals as it would create unpredictable patterns of activity. You have a large sample of single-unit recordings. Can you verify the results remain the same?

Line 221: Should state muscles sampled, and if any repeats from same muscles. Yes this is not interesting information, but important if someone wants to replicate your study.

Figure 2: the inset panels highlight that initial movement directions were highly biased towards 2 or 3 directions, particularly JAD. How does this non-uniform direction of initial movements affect the analysis? For example, you could reduce the number of movement directions to be even more biased to see if that affects the results.

While condition-independent terms are clearly less common for muscles than cells, these terms seem to be more than I would have expected. I'd like to see the analysis from Fig 5 completed for the muscle sets for comparison purposes to support their claims that muscles have little or no CIS (line 795). Not sure these signals can be assumed to reflect co-contraction signals. If so, then some of the condition-independent information in motor cortex may also be related to co-contraction.

Line 725-227: I have to say I'm either surprised or confused about the statement that 50% of neurons with the highest baseline converts into firing rates from 2 to 88 spikes/s (median 8 spikes/s) at target onset. These neurons with firing rates below 8 (half of cells) are going to be fairly constrained to only showing firing rate increases.

Minor

Line 173 and 413: The removal of long RTs and non-bell shaped velocity profiles mentioned twice so perhaps keep at one location.
